# Nutrition in palliative care at the end of life: Bibliometric and network analysis until 2024

**DOI:** 10.1097/MD.0000000000043381

**Published:** 2025-07-18

**Authors:** Carlos Álvaro Araújo, Rauany Fiuza Braga Pires de Melo, Geisa Sant Anna, Fábio Ferreira Amorim

**Affiliations:** a Graduation Program in Health Sciences, University of Brasília (UnB), Brasília, Brazil; b Intensive Care Unit, Hospital Sírio Libanês Brasília, Brasília, Brazil; c Graduation Program in Health Sciences of School Health Sciences, Escola Superior de Ciências da Saúde (ESCS), Brasília, Brazil.

**Keywords:** diet, food, and nutrition, food, nutrition therapy, palliative medicine, terminal care

## Abstract

**Introduction:**

Food consumption is not only driven by a desire for nutrients and satiety but also a fundamental element of human interaction. This study aimed to comprehensively analyze bibliometric characteristics, collaboration networks, points of interest, and research trends, providing insights into the current landscape of publications on nutrition in palliative care at the end of life (PC-EOL).

**Methods:**

The Web Wof Science Core Collection database was screened for studies addressing PC-EOL, published until December 2024. From the search results, we performed statistical descriptions of the year of publication, study type, authors, country, institution, keywords, scientific journal, citations, and disciplinary (Web of Science category). VOSviewer 1.6.19 (visualization of similarities), Microsoft Office Excel 16.78 for Mac, BibExcel, and the Bibliometrix R package, along with the Biblioshiny tool, were used for data analysis.

**Results:**

Three hundred fourteen articles from 48 countries were included. Annual publications increased from 4 in 1994 to a peak of 35 in 2022 but declined over the past 2 years, reaching 16 in 2024. Most articles were from the United States, but the United Kingdom had the highest influence on the network. Keywords were stratified into 9 clusters: “Comprehensive End of Life Care,” “Ethics and Decision-Making in End-of-Life,” “Eating Disorders and Assisted Dying,” “Palliative and Long-Term Care,” “Palliative and Long-Term Care,” “Advanced Nutritional Support,” “Critical Care,” “Family and Pediatric Care,” “Advanced Cancer Management,” and “Gastrointestinal Obstruction.” Trend topic analysis suggests an increase in terms related to patients’ refusal of nutrition/artificial nutrition, ethics, family, bereavement, and eating disorders, shifting from prior main themes, such as withdrawal/withholding life-sustaining therapy in advanced cancer/dementia. Among the top 20 cited articles, although all addressed nutrition in PC-EOL, it was the core element in only 9.

**Conclusion:**

The number of articles published annually remains limited, with low levels of collaboration among countries and authors. Few studies have examined the symbolic and relational meanings of food in terminal illness from the perspectives of patients and their families, dimensions crucial to truly person-centered care. Future research should prioritize the lived experiences, cultural contexts, and ethical concerns of those directly affected.

**Key points**

- Nutrition in palliative care continues to pose challenges, as existing studies are frequently fragmented and lack comprehensive synthesis.
- Patient and family involvement in end-of-life (EOL) nutritional decision-making has increased in prominence.
- A more nuanced understanding of the patient-family dynamic in EOL nutritional decision-making is needed.

## 1. Introduction

Nutrition encompasses fundamental physiological necessities, carrying psychological, social, and symbolic significance. At the end of life (EOL), there is a progressive decline in the ability to consume food or beverages orally. In this respect, terminally ill patients should be frequently assisted in consuming food as long as they desire, as an integral part of fundamental care.^[1–3]^ Conversely, some patients refuse nutrition if there is no chance of a cure.^[1]^ As an intervention, the commencement, cessation, and withholding of nutrition support necessitate sound medical and ethical justifications.^[3–5]^

When considering nutritional support for a dying patient, healthcare professionals (HCPs) must recognize the transition when the body begins to shut down due to disease and the dying process, at which point nutrition may no longer provide benefits.^[6]^ Besides, patients may not tolerate tubes well due to discomfort, requiring restraint to prevent accidental removal. Tube feeding also entails the adverse consequences of depriving patients of the taste and texture of food, as well as social and human contact associated with being hand-fed.^[3,7,8]^ Nevertheless, psychological distress may arise if cognitive deterioration prevents a patient from expressing their wishes, with dehydration and fasting possibly contributing to agitated delirium or terminal restlessness.^[1]^

The involvement of patients and their families in treatment decisions regarding nutrition support at the EOL has gained increasing importance. However, the concepts associated with this dynamic are seldom understood and need improvement.^[9]^ For instance, family members in EOL situations frequently clash with the dying patient’s prior wishes and HCPs to establish appropriate nutritional care.^[10–12]^ Indeed, the data from studies on nutrition in palliative care (PC) patients are predominantly fragmented and lacking synthesis. In 2014, the latest update of a Cochrane Collaboration review on the use of artificial nutrition in adult patients with PC concluded that there was insufficient evidence to formulate practice guidelines.^[13]^

Understanding and synthesizing all available data related to nutrition in palliative care at the end of life (PC-EOL) is critical for HCPs to address key challenges and identify existing research gaps. To bridge these gaps, this study aims to comprehensively analyze bibliometric characteristics, collaboration networks, points of interest, and research trends, providing insights into the current landscape of publications on nutrition in PC-EOL.

## 2. Materials and methods

### 2.1. Study type

This study presents a bibliometric and network analysis of scientific articles addressing PC-EOL, published up to December 2024. The last literature search was conducted on February 2, 2025, without language restrictions, following the Preliminary guideline for reporting bibliometric reviews of the biomedical literature proposed by Montazeri et al.^[14]^

### 2.2. Eligibility criteria

The guiding question for this study was: What are the bibliometric characteristics, network collaborations, focal points, trends, and gaps in manuscripts published in scientific journals addressing nutrition in PC-EOL?

The population, context, and concept framework served as the guiding criterion:

- Population (P): PC-EOL patients,
- Concept (C): Nutrition in PC, and
- Context (C): Hospital and hospice.

Inclusion criteria included experimental, quasi-experimental, and observational studies with quantitative, qualitative, and mixed methods. Studies were considered if they met the specified inclusion criteria and did not meet any exclusion criteria. Narrative, scoping, and systematic reviews were also included in the analysis. Studies addressing PC patients involving curative therapies and conference abstracts were excluded. There were no language restrictions.

Table S1, Supplemental Digital Content, https://links.lww.com/MD/P429 outlines the inclusion and exclusion criteria based on the population, context, and concept framework.

### 2.3. Studie’s identification

#### 2.3.1. Database

Data were extracted from the Web of Science Core Collection (WOSCC), the most widely used database in bibliometric analyses, which provides a structured format for citation determination and network analysis.^[14,15]^

#### 2.3.2. Search strategy

An advanced search in the WOSCC was conducted on February 2, 2025, encompassing publications with a search limit up to December 31, 2024. The search strategy is detailed below:

TS=((Terminal Care OR Palliative Care OR Palliative Medicine OR Terminally Ill OR Resuscitation Orders OR Hospice Care) AND (Nutrition Therapy OR Diet OR Parenteral Nutrition, Total OR Protein Hydrolysates OR Parenteral Nutrition OR Parenteral Nutrition Solutions OR Fat Emulsions, Intravenous OR Enteral Nutrition OR Diet, Food, and Nutrition OR Eating OR diet therapy OR Food OR Food, Formulated OR Dietary Services OR Dietetics OR Dietary Supplements) AND (Food Service, Hospital OR Intensive Care Units OR Critical Care OR Hospices OR Inpatients OR Hospitals, General OR Hospital Units OR Patients’ Rooms OR Hospitalization OR Oncology Service, Hospital OR Inpatient Care Units OR Beds)).

#### 2.3.3. Extraction and selection

Following the WOSCC search, data about the retrieved studies were selected as “Complete Record and Cited References” and extracted from the WOSCC database for further analysis.

The extracted file containing data on the identified articles was transferred to the Rayyan software (https://www.rayyan.ai). Two reviewers independently assessed titles, abstracts, and, subsequently, full texts for eligibility. A third reviewer resolved disagreements.

### 2.4. Variables and database adjustment

The following variables were assessed: year of publication, study type, authors, country, institution, keywords, scientific journal, citations, and disciplinary (WOS category).

Two reviewers independently classified the study type and harmonized keywords and institutional names to ensure consistency across the database. In cases of disagreement, a third reviewer was consulted. Synonyms and institutional name variations were reconciled, and keywords were added to articles lacking author-defined terms based on full-text review.

### 2.5. Data synthesis

Bibliometrics is one of the primary methodologies for objectively measuring the impact of academic publications within a specific field.^[16,17]^ It encompasses mathematical modeling, statistical analysis, and social network analysis to explore the knowledge structure, critical research junctures, academic communities, and the current status and future trends related to a particular theme.^[17,18]^

The Bibliometrix R package, along with the Biblioshiny tool, VOSviewer 1.6.19 (visualization of similarities), Microsoft Office Excel 16.78, and BibExcel, were used for data analysis.

#### 2.5.1. Bibliometric characteristics

VOSviewer 1.6.19, BibExcel, and Bibliometrix R package, combined with the Biblioshiny tool, were used to analyze bibliometric data, including study design, authors, years of publication, scientific journals, citations, and the *H*-index, *G*-index, and *M*-index of authors and journals, as well as the countries and institutions involved. The corresponding figures were generated using Microsoft Office Excel 16.78. Additionally, the 2-year moving average and the exponential regression model for annual publication counts were calculated and visually represented using Microsoft Excel 16.78.

The Bradford law, also known as the law of dispersion, was used to establish the core scientific journals with publications related to the PC-EOL.^[19]^

#### 2.5.2. Collaboration networks

Collaboration networks among countries, institutions, and authors were established and graphically represented using the Bibliometrix R package with the Biblioshiny tool and VOSviewer 1.6.19. In each network, nodes represent institutions, countries, or authors, while connections indicate collaboration among the interconnected nodes. The thickness of the links portrays the strength of these connections subsequently, the influence of each node and the strength of its interactions within the entire social network were evaluated using social network analysis.^[17,20,21]^

To evaluate node influence, 3 centrality measures were employed: betweenness centrality (BC), closeness centrality (CC), and PageRank. A higher BC value denotes a more vital ability to govern the entire flow of collaboration within the network, placing the node strategically at the network’s center. On the other hand, CC measures the distance between nodes, and a smaller distance corresponds to a higher CC value, indicating that the node holds a pivotal position throughout the network.^[17,22]^ PageRank operates on the premise that nodes of significance are likely linked to other important nodes. Therefore, a node’s PageRank is determined by the quantity and quality of connections pointing to it. If numerous relevant nodes interconnect with a particular node, that node receives a higher score. Nodes with higher scores in their PageRank contribute more significantly to the score of the target node.^[23]^

### 2.6. Points of interest

VOSviewer 1.6.19, BibExcel, and Bibliometrix R package, combined with the Biblioshiny tool, were used to analyze data regarding the overall frequency of each keyword and disciplinary area. The corresponding figures were generated using Microsoft Office Excel 16.78. Subsequently, Price’s law was applied to calculate the minimum frequency value (*M*) for a keyword to be considered relevant in defining the research hotspot within the published articles.^[24]^ In this context, *M* was defined as the square root of the frequency of the most occurring keyword (N_max_) multiplied by 0.7498.^[17]^

Co-occurrence among keywords was established and graphically represented using the Bibliometrix R package with the Biblioshiny tool. Co-occurrence among disciplinary areas was constructed in VOSviewer 1.6.19, employing the visualization of similarities method.^[17,20,21]^

### 2.7. Research trends

The network chronological overlay of keywords was constructed and visualized using VOSviewer 1.6.19.

Using the degree of centrality and thematic development metrics, strategic thematic maps of articles published between 1994 and 2016 and those published between 2017 and 2024 were compared and visualized with the Bibliometrix R package via the Biblioshiny interface. The resulting figure was subsequently adjusted and optimized for resolution and clarity using an AI-based enhancement tool.^[25,26]^

The thematic strategic map is a graphical representation that displays sets of keywords arranged in 4 quadrants, defined by an *x*-axis (density or degree of development) and a *y*-axis centrality or degree of relevance):

- First quadrant (top-right): Central and developed, associated with motor themes.
- Second quadrant (top-left): Central and undeveloped, associated with basic and transversal themes.
- Third quadrant (bottom-left): Peripheral and developed, associated with developed and isolated themes.
- Fourth quadrant (bottom-right): Peripheral and undeveloped, associated with emerging or declining themes.

Figure S1, Supplemental Digital Content, https://links.lww.com/MD/P430 shows the Instruments/Software used for the bibliographic and network analysis.

## 3. Results

### 3.1. Bibliometric characteristics

#### 3.1.1. Publication summary

Based on the search strategy, 511 articles were retrieved until December 2024. Of these, 197 articles were excluded: 146 due to the incorrect population, 44 due to the incorrect intervention, 6 due to the incorrect publication type, and 1 due to duplication. Consequently, 314 articles addressing nutrition in PC-EOL were included in the bibliometric review, Figure S2, Supplemental Digital Content, https://links.lww.com/MD/P430.

The included articles were written by 1504 authors from 48 countries and published in 176 journals with a mean of 5.3 coauthors/document and 23.6 citations/doc, Table S2, Supplemental Digital Content, https://links.lww.com/MD/P429.

**Figure 1.**
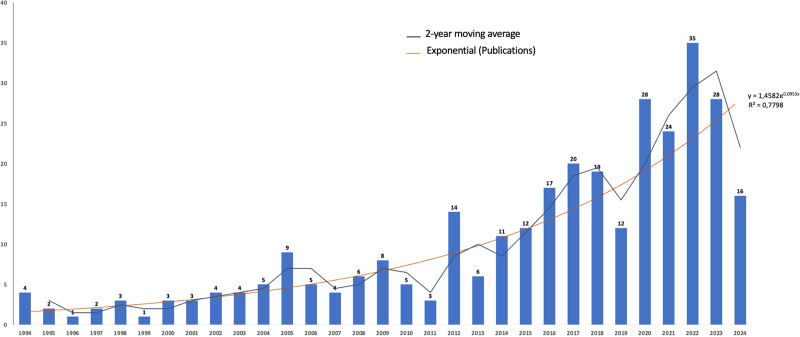
Annual publications addressing nutrition in palliative care at the end of life until 2024.

Figure 1 and Table S3, Supplemental Digital Content, https://links.lww.com/MD/P429 show the articles addressing nutrition in PC-EOL published by year until 2022. The oldest article addressing nutrition in PC-EOL was published in 1994. The number of annual publications increased from 4 in 1994 to a peak of 35 in 2022 but declined over the past 2 years, reaching 16 in 2024. The 2-year moving average for the period from 2023 to 2024 was 22.0.

**Figure 2.**
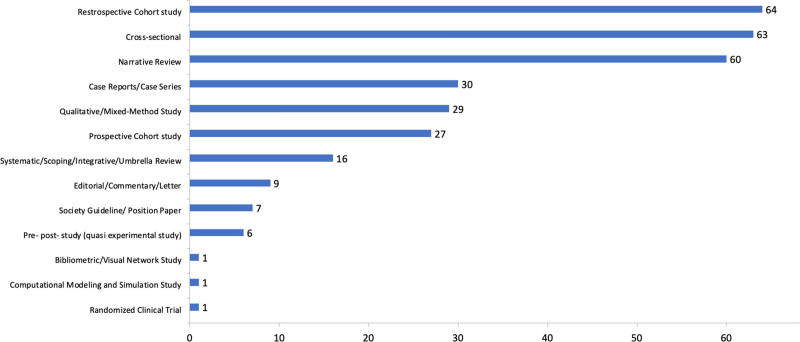
Publications addressing nutrition in palliative care at the end of life until 2024, according to the study design.

Figure 2 and Table S4, Supplemental Digital Content, https://links.lww.com/MD/P429 show the articles addressing nutrition in PC-EOL according to the study design. The most frequent study designs were retrospective cohort studies (n = 64, 20.4%), cross sectional studies (n = 63, 20.1%), and narrative reviews (n = 60, 19.1%). There were only 7 (2.2%) interventional studies, comprising 6 quasi-experimental studies and 1 randomized clinical trial, all of which were published from 2017 onwards.

#### 3.1.2. Countries´ productivity

The published articles addressing nutrition in PC-EOL were from 48 countries. Table S5, Supplemental Digital Content, https://links.lww.com/MD/P429 shows the top 25 countries ranked by the number of publications. The United States (US) was the most productive country, with 125 (39.8%) articles, followed by the United Kingdom (UK; n = 37, 11.8%), Germany (n = 21, 6.7%), Japan (n = 19, 5.7%), and China (n = 18, 5.7%). Regarding regions among the top 25 countries ranked by the number of publications, Europe has 14 countries with publications, Asia has 7, North America has 2, and Oceania, Africa, and South America have 1.

Table S6, Supplemental Digital Content, https://links.lww.com/MD/P429 shows the publications addressing nutrition in PC-EOL by first authors’ countries. The US accounted for the highest number of first authors (n = 114, 36.3%), followed by the UK (n = 26, 8.3%), China (n = 17, 5.4%), Japan (n = 17, 5.4%), and Germany (n = 15, 4.8%).

Table S7, Supplemental Digital Content, https://links.lww.com/MD/P429 shows the publications addressing nutrition in PC-EOL by corresponding authors’ countries. The US had the highest number of corresponding authors (n = 117, 37.3%), followed by the UK (n = 26, 8.3%), China (n = 17, 5.4%), Japan (n = 17, 5.4%), and Germany (n = 14, 4.5%).

#### 3.1.3. Author’s productivity

A total of 1504 authors published articles addressing nutrition in PC-EOL. Among them, 1381 wrote 1 article, 98 wrote 2, 15 wrote 3, 5 wrote 4, and 5 wrote 5. Table S8, Supplemental Digital Content, https://links.lww.com/MD/P429 shows the top 10 authors ranked by the number of publications. Mitchell SL, Bruera E, Morita T, Quill TE, Yager J, and Morita T were the most productive authors, publishing 5 articles. Mitchell SL, Bruera E, Quill TE have the highest *H*-index, each with a score of 5.

#### 3.1.4. Journals´ distribution

One hundred sixty-six journals published articles addressing nutrition in PC-EOL. Figure S3, Supplemental Digital Content, https://links.lww.com/MD/P430 shows the top 13 journals ranked by the number of publications. According to Bradford’s law, the core journals on nutrition in PC-EOL are the 10 journals that published 6 or more articles: Journal of Pain and Symptom Management Medicine (n = 20, 6.4%), Journal of Palliative Medicine (n = 18, 5.7%), American Journal of Hospice & Palliative Medicine (n = 12, 3.8%), Palliative Medicine (n = 10, 2.3%), BMJ Supportive & Palliative Care (n = 10, 3.2%), Supportive Care in Cancer (n = 9, 2.9%), BMC Palliative Care (n = 8, 2.5%), Journal of Palliative Care (n = 8, 2.5%), Journal of Eating Disorders (n = 6, 1.9%), and Journal of the American Geriatrics Society (n = 6, 1.9%).

Journal of Pain and Symptom Management, with 20 publications (*H*-index: 10, *G*-index: 18, *M*-index: 0.417, and total citations: 359), was the most influential, followed by Journal of Palliative Medicine, with 18 publications (*H*-index: 9, *G*-index: 14, *M*-index: 0.450, and total citations: 203), indicating that these journals are the highly recognized publications in the cross field addressing nutrition in PC-EOL, Table S9, Supplemental Digital Content, https://links.lww.com/MD/P429.

#### 3.1.5. Institutions´ productivity

Seven hundred twenty-two institutions published articles addressing nutrition in PC-EOL. Figure S4, Supplemental Digital Content, https://links.lww.com/MD/P430 and Table S10, Supplemental Digital Content, https://links.lww.com/MD/P429 show the top 14 institutions ranked by the number of publications addressing nutrition in PC-EOL. The Harvard University (n = 8) and University of Colorado (n = 8) were the most productive institutions. Among the top 14 institutions, 11 were from the US, 1 from Canada, 1 from Japan, and 1 from Switzerland.

#### 3.1.6. Most frequently cited articles

The articles had 7407 citations, a mean of 23.6 citations/article. Table S11, Supplemental Digital Content, https://links.lww.com/MD/P429 shows the top 20 articles addressing nutrition in PC-EOL in terms of citations.^[27–46]^ Mitchell et al^[27]^ is the article with the highest citations (n = 877) worldwide, followed by McCann et al^[28]^ with 336 citations, Eddy et al^[29]^ with 281 citations, and Nitenberg et al^[29]^ with 271 citations. Although ranking sixth in citations worldwide, Ganzini et al^[31]^ had the highest impact in terms of local citations (n = 20), followed by McCann et al^[28]^ with 15 citations, and Druml et al^[32]^ with 14 citations.

### 3.2. Collaboration networks

#### 3.2.1. Countries’ collaboration

**Figure 3.**
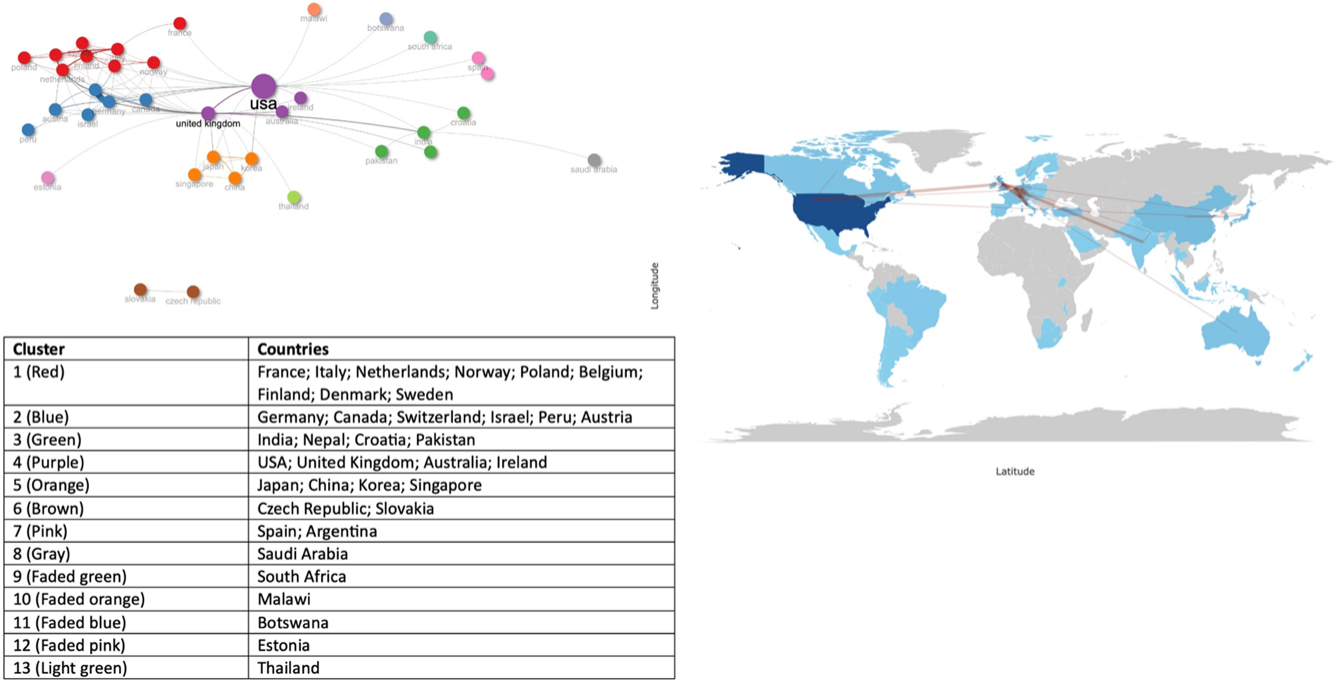
Countries’ collaboration on publications addressing nutrition in palliative care at the end of life until 2024.

Figure 3 and Table S12, Supplemental Digital Content, https://links.lww.com/MD/P429 show the collaboration network analysis among countries. The UK has the highest BC: 254.479, with a PageRank of 0.115 and a CC of 0.223, indicating the most robust ability to control the information flow in the entire network. The US has the second highest influence (BC: 233.504, PageRank: 0.097, and CC: 0.021). In addition, India (BC: 42.908, PageRank: 0.038, and CC: 0.016), Italy (BC: 18.031, PageRank: 0.040, and CC: 0.017), and Switzerland (BC: 14.701, PageRank: 0.018, and CC: 0.065) have a denoting cooperation with other countries.

Most publications are single-country publications (SCPs), even in countries that demonstrated higher levels of collaboration within the network. Among the 114 publications with a first author from the US, 106 were SCPs, and 8 were multiple-country publications (MCPs). Switzerland is the second country ranked in MCPs, with 5 MCPs and 3 SCPs, Table S6, Supplemental Digital Content, https://links.lww.com/MD/P429.

#### 3.2.2. Institutions´ collaboration

**Figure 4.**
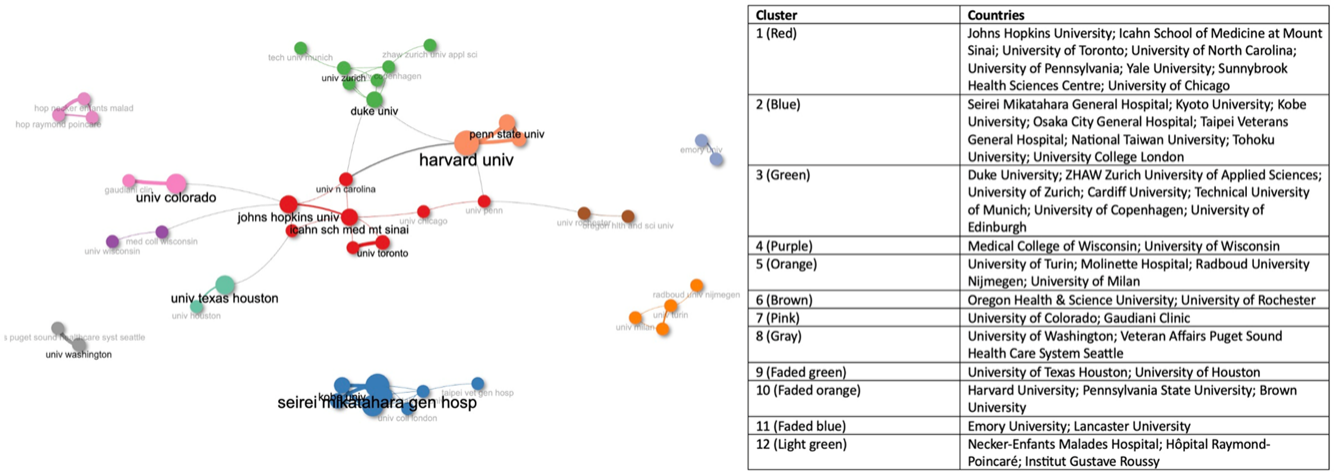
Institutions’ collaboration on publications addressing nutrition in palliative care at the end of life until 2024.

Figure 4 and Table S13, Supplemental Digital Content, https://links.lww.com/MD/P429 show the collaboration network analysis among the 50 most productive institutions. The University of North Carolina has the highest BC (121.000) with a PageRank of 0.025 and CC of 0.018, followed by Duke University (BC: 114.000, PageRank: 0.031, and CC: 0.016) and Johns Hopkins University (BC: 104.500, PageRank: 0.035, and CC: 0.016), showing most solid cooperation with other institutions in the network.

#### 3.2.3. Authors´ collaboration

Figure S5, Supplemental Digital Content, https://links.lww.com/MD/P430 shows the collaboration network among authors with 2 or more publications. The collaboration between authors is weak, as all authors have BC below 10.

### 3.3. Points of interest

Eight hundred twenty-two author keywords were extracted from the articles. After merging synonyms and duplications, 767 keywords were obtained. Among them, 495 appeared once (64.5%). Table S14, Supplemental Digital Content, https://links.lww.com/MD/P429 shows the high frequency authors’ keywords. According to Price’s law, the 46 authors’ keywords with a frequency of 11 or greater are considered high frequency keywords. Top 10 keywords by frequency are “palliative care” (n = 155), “cancer” (n = 99), “end of life” (n = 79), “hospice” (n = 68), “nutrition” (n = 68), “end of life care” (n = 51), “advanced cancer” (n = 31), hospice care” (n = 28), “ethics” (n = 25), “ and “parenteral hydration” (n = 23).

**Figure 5.**
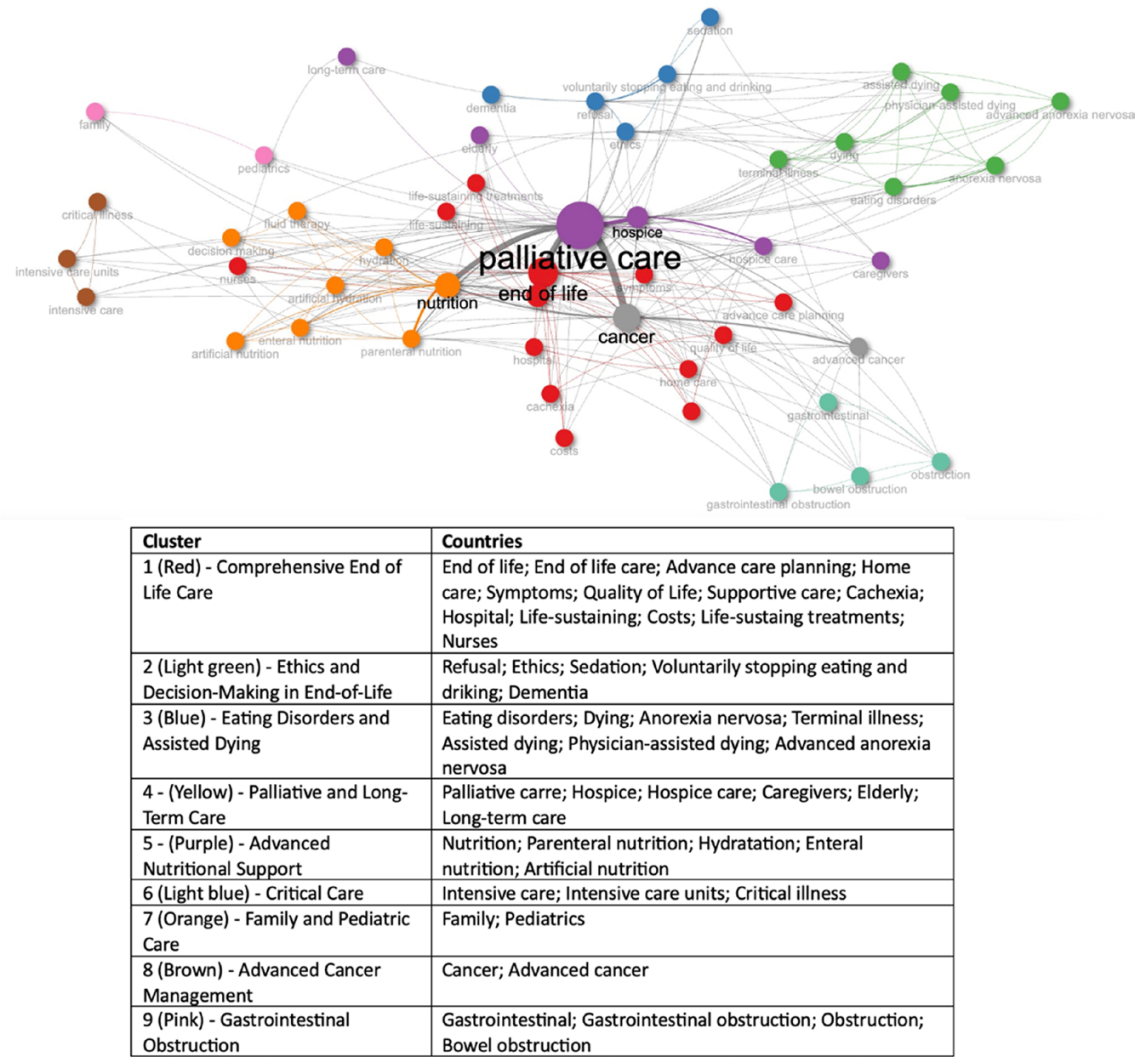
Authors’ keywords network visualization in terms of co-occurrence on publications addressing nutrition in palliative care at the end of life until 2024.

Figure 5 shows the most cited authors’ keyword co-occurrence on publications. Four clusters were formed, representing the research hotspots addressing nutrition in PC-EOL between 1994 and 2024. Cluster 1 (“Comprehensive End of Life Care”) is the largest category and has 13 keywords, Cluster 2 (“Ethics and Decision-Making in End-of-Life”) has 5, Cluster 3 (“Eating Disorders and Assisted Dying”) has 7, Cluster 4 (“Palliative and Long-Term Care”) has 6, Cluster 5 (“Advanced Nutritional Support”) has 5, Cluster 6 (“Critical Care”) has 3, Cluster 7 (“Family and Pediatric Care”) has 2, Cluster 7 (“Advanced Cancer Management”) has 2, and Cluster 9 (“Gastrointestinal Obstruction”) has 3.

Published articles addressing nutrition in PC-EOL were classified into WOS categories. Figure S6, Supplemental Digital Content, https://links.lww.com/MD/P430 shows the distribution of the top 10 disciplinary areas according to WOS categories. “Health Care Sciences & Services” was the most frequent category (n = 104, 33.1%), followed by “Medicine, General & Internal” (n = 64, 20.4%), and “Oncology” (n = 34, 10.8%).

Figure S7, Supplemental Digital Content, https://links.lww.com/MD/P430 shows the WOS categories’ co-occurrence in publications. Ten clusters were formed with 61 WOS categories that had connections. These 9 clusters represent the disciplinary area hotspots addressing nutrition in PC-EOL between 1994 and 2024. Using the largest node in each research hotspot as the cluster name, “Nutrition & Dietetics “ (Cluster 1) has 9 categories, “Ethics” (Cluster 2) has 8, “Oncology” (Cluster 3) has 7, “Health Care Sciences & Services” (Cluster 4) has 5, “Pediatrics/Obstetrics & Gynecology” (Cluster 5) has 4, “Public, Environmental & Occupational Health” (Cluster 6) has 4, “Rehabilitation” (Cluster 7) has 3, “Surgery” (Cluster 8) has 3, “Health Policy & Services” (Cluster 9) has 3, and “Geriatrics & Gerontology” (Cluster 10) has 2. “Health Care Sciences & Services” has the highest centrality and connection compared to other clusters.

Table S15, Supplemental Digital Content, https://links.lww.com/MD/P429 shows the viewpoint of the top 20 cited articles addressing nutrition in PC-EOL in terms of citations. Table S15, Supplemental Digital Content, https://links.lww.com/MD/P429 shows the viewpoint of the top 20 cited articles addressing nutrition in PC-EOL in terms of citations. Among the top 20 cited articles, 5 comprehend the authors’ keyword cluster theme “Comprehensive End of Life Care,” 5 “Ethics and Decision-Making in End-of-Life,” 5 “advanced cancer Management,” 3 “Gastrointestinal obstruction,” 2 “ Ethics and Decision-Making in End-of-Life,” and 1 “Eating Disorders and Assisted Dying.” Although all articles addressed nutrition in PC-EOL in their content, it was the core element in only 9.

### 3.4. Research trends

**Figure 6.**
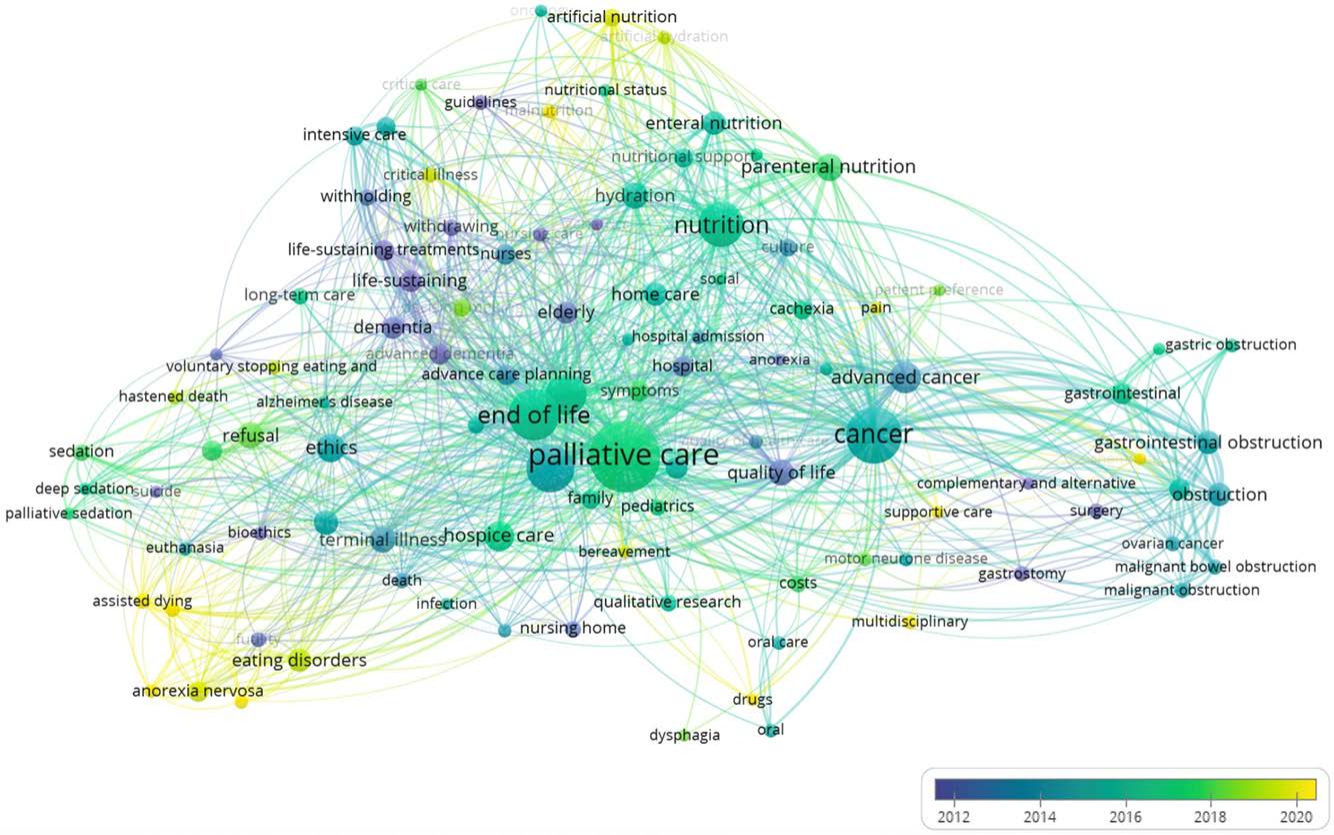
Authors’ keywords overlay visualization by year on publications addressing nutrition in palliative care at the end of life until 2024.

Figure 6 and Figure S8, Supplemental Digital Content, https://links.lww.com/MD/P430 show the historical evolution of the occurrence of authors’ keywords in publications. The trend topic analysis suggests an increase in terms related to patients’ refusal of nutrition and artificial nutrition, assisted dying, drugs, mental illness, anorexia nervosa, and eating disorders in the last years, shifting from the main themes, such as guidelines, geriatrics, and life-sustaining treatment in advanced cancer and dementia.

**Figure 7.**
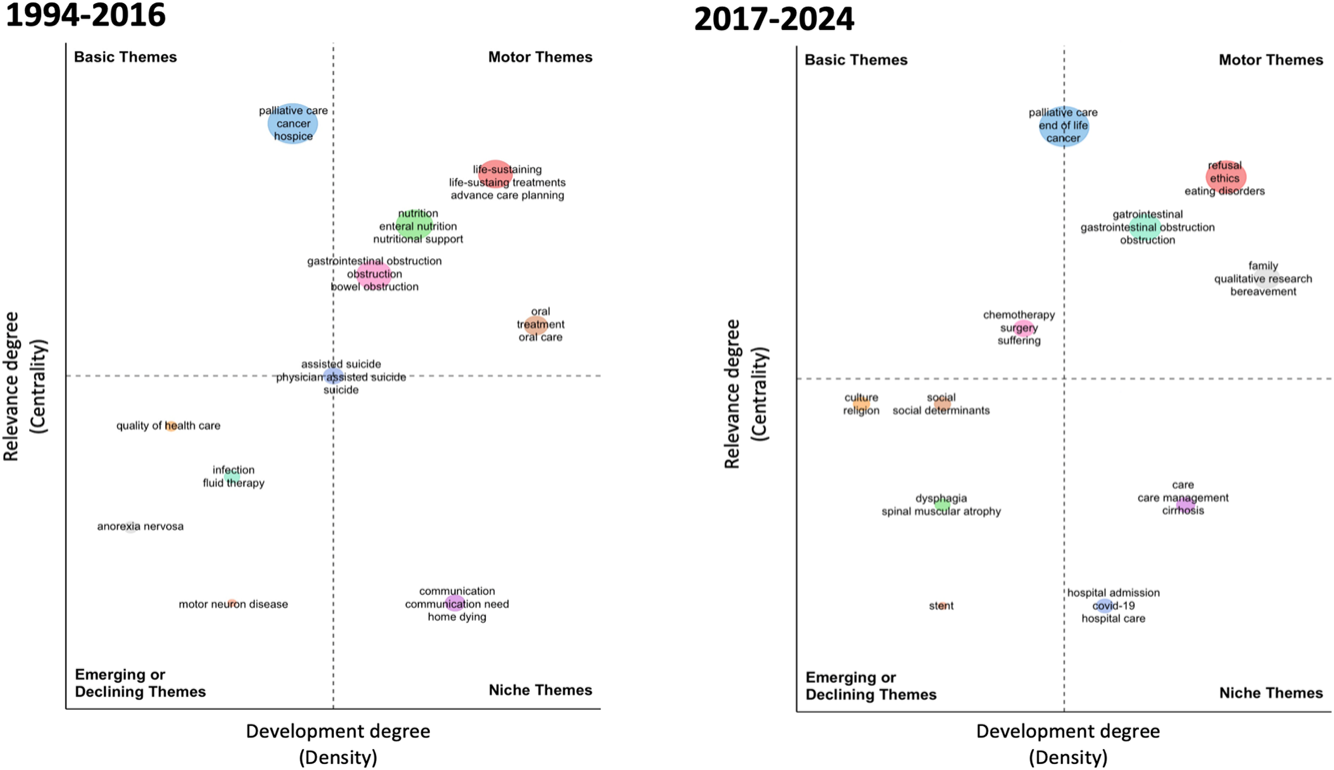
Authors’ keywords strategic thematic map on publications addressing nutrition in palliative care at the end of life from 1994 to 2016 and 2017 to 2024.

Figure 7 shows the strategic thematic map of publications addressing nutrition in PC-EOL from 1994 to 2016 and 2017 to 2024. In the motor themes, topics such as life-sustaining treatments and advance care planning, which had high centrality (relevance) and density (development) from 1994 to 2016, shifted from 2017 to 2024 toward themes focusing on PC in patients’ refusal of nutrition/artificial nutrition, ethics, family, bereavement, and eating disorders, the latter having been an emerging theme in the earlier period. Notably, patients’ refusal of nutrition and artificial nutrition also appeared as motor themes with high centrality, reflecting a growing research focus on ethical dilemmas and patient autonomy in PC-EOL. Meanwhile, PC and nutrition in advanced cancer remained a high-centrality, low-density theme in both periods, indicating its continued relevance but relatively limited development in the literature. Additionally, goals of care and menu optimization, which were absent in the thematic map from 1994 to 2016, emerged with high density from 2017 to 2024, positioning them between niche and motor themes. New niche themes emerged from 2017 to 2024, including multidisciplinary and oral care in motor neuron disease and PC, as well as education in advanced heart failure, suggesting an expanding research focus on neurological and cardiovascular conditions within PC-EOL.

## 4. Discussion

### 4.1. Bibliometric characteristics

The evolution of scientific publications over time reflects not only the development of a field but also shifting research priorities.^[47]^ Our bibliometric and network analysis reveals that, despite a temporary growth in publications related to nutrition in palliative and end-of-life care (PC-EOL) up to 2024, the overall volume remains markedly low. This finding contrasts with the sharp and sustained increase in broader PC literature worldwide, which is projected to reach 2470 publications in 2025.^[48]^

Our study also highlights the predominance of retrospective and cross-sectional designs, underscoring the persistent lack of high-quality, prospective evidence in the field. This issue is not merely a methodological shortcoming but rather a structural and ethical challenge inherent to conducting research with patients at the EOL.^[49–51]^ A global bibliometric analysis of supportive and palliative oncology revealed that only 6% of publications were randomized clinical trials,^[49]^ and even among these, many demonstrated methodological weaknesses.^[50]^ Several factors contribute to this gap, including ethical dilemmas, difficulties in recruiting and retaining terminally ill patients, and logistical constraints.^[3,52]^ In addition, research in PC remains significantly underfunded compared to studies focused on curative or preventive interventions for life-limiting conditions.^[52–55]^ These challenges point to the urgent need for innovative research designs and ethical frameworks that enable the production of robust, clinically meaningful evidence while safeguarding the dignity and rights of this vulnerable population.

The US was the most productive country, followed by the UK. The US has a rich history of PC development, and in recent times, PC services have rapidly expanded within its healthcare system.^[56]^ It is essential to note that all top 10 countries regarding publication were high-income and upper-middle-income countries, and only 1 from South America (Brazil) and 1 from Africa (South Africa) are among the top 26 countries. The significant number of articles from North America and Europe and the lack of publications in low-middle- and low-income countries was also observed in other bibliometric analysis studies that evaluate PC patients.^[48,55,57,58]^ Approximately 56.8 million people require PC annually worldwide, including 31.1 million before and 25.7 million near the EOL, and only 12% of patients who need PC receive it.^[59]^ Notably, most adults in need of PC (76%) live in low-middle-income countries, and the highest proportion are in low-income countries, showing a need for studies including these populations also addressing its social and cultural peculiarities, which accompanies the substantial gap in funding and availability of PC amongst country groups by income.^[55,59–64]^

Among the top 10 journals in terms of the number of publications addressing nutrition in PC-EOL, most fall under the “Palliative Care” subject, with none falling under the “Nutrition and Dietetics” subject. This finding highlights the insufficient emphasis on this topic in nutrition and dietetics, as well as multidisciplinary journals, although most of the highest-cited articles assessing nutrition in PC-EOL were published in journals not directly dedicated to PC publications. Many patients with PC experience a reduction in oral intake during their illness, highlighting the crucial role of nutritional support in their overall care. This reduction most frequently occurs during the terminal phase, characterized by a decline in consciousness, rendering the patient less amenable to oral nutritional intake. Previous systematic reviews and guidelines assessing nutrition in patients with PC have consistently observed that the available evidence falls short of providing substantive guidance for formulating comprehensive practice guidelines. This aspect underscores the need for further research and increased multidisciplinary attention to nutrition in PC-EOL.^[2,9,13,32,34,51]^

### 4.2. Collaboration networks

Regarding the countries’ collaborations, although the US has the highest number of published articles, the UK has the highest ability to control the information flow in the entire network. The UK is a world leader in PC-EOL care, significantly advancing EOL care practices worldwide.^[48,53,65]^ Certainly, PC holds a prominent position in the priorities of the UK National Health Service, with the development of numerous national guidelines in recent years. This commitment extends to founding researchers to deliver evidence-based studies to shape national policies.^[53,66,67]^

Most publications are SCPs, indicating a need for collaboration efforts among nations, highlighting that development strategies for global collaboration in this field are a priority, particularly in searching for the best scientific evidence and reducing inequities among those requiring EOL care.^[53,59]^

The will, determination, and innovation of collaborative organizations will enhance PC-EOL.^[66]^ Not surprisingly, among the top 14 leading research institutions in terms of publication numbers, eleven are based in the US. All 3 authors with the highest *H*-index are from the US. Findings that confirm the relevance of the US in the field of PC enhancement were observed in prior PC bibliometric analysis studies.^[48,57,58]^ However, similar to the patterns observed in a bibliometric analysis study evaluating research on perinatal PC worldwide, numerous institutions lacked collaboration with other institutions. When present, most collaborations were restricted to institutions within the same country or exhibited evident geographic clustering characteristics, with little to no collaboration between these clusters, a pattern also observed in the collaboration between the authors. This phenomenon may be attributed to the conducive factors of geographic proximity and cultural affinity, which make it the more accessible foundation for international cooperation and collaboration within the clusters.^[58]^ An additional explanation is that most sponsoring or funding institutions concentrate on research aligned with their priorities within a specific country or region.^[48,68,69]^

### 4.3. Points of interest

Regarding the disciplinary area’s hotspots according to the WOS categories, “Health Care Sciences & Services” is the most frequent category, focusing on the organization of health systems and services, as well as aspects of nutrition in end-of-life care (EOL), with less emphasis on addressing individual patient and family needs.^[51,53,64]^ A paramount example of this trend was the global dissemination of EOL pathways without compelling evidence of their impact on patients, families, and professional practice, showing a bias toward biomedical aspects.^[70–73]^ Notably, in England, implementing The Liverpool Care Pathway for the Care of the Dying Patient, used from the late 1990s until 2014, was associated with reports of withdrawal nutrition and hydration based on “best interests” rationale without explanation or consultation for patients and their relatives.^[3,54,74]^

The marginal representation of studies addressing nutrition in PC-EOL, despite the sustained growth of the broader PC literature worldwide, highlights that nutritional care remains a peripheral concern within the palliative field.^[48]^ A notable contribution of our study is the quantification of this underrepresentation: among the 20 most cited articles in PC-EOL addressing nutrition, fewer than half consider it as a central research focus.^[27–46]^ For instance, the most cited study, published by Mitchell et al,^[27]^ reported that 85.8% of patients with advanced dementia experienced eating problems, thereby reinforcing the centrality of nutrition in EOL dementia care. The second most cited study, published by McCann et al,^[28]^ found that minimal nutrition and hydration were often sufficient to alleviate discomfort in terminally ill patients, supporting a conservative and comfort-oriented approach. The third most cited study, published by Eddy et al,^[29]^ highlighted that recovery from anorexia nervosa continued even after a decade of illness, challenging the applicability of PC in long-term eating disorders. This finding highlights a thematic and disciplinary gap that persists across both PC and nutrition science, and it is further reflected in the absence of nutrition-focused journals among the top publication venues for this topic.

### 4.4. Research trends

Although PC and nutrition in advanced cancer have high centrality and density in the strategic thematic maps of publications from 1994 to 2016 and 2017 to 2024, the authors’ keywords and trend topic analysis suggest an increase in terms related to patients’ refusal of nutrition/artificial nutrition, ethics, family bereavement, and eating disorders in the last years, shifting from the prior main themes, such as withdrawal and withholding life-sustaining therapy in advanced cancer and dementia. In recent years, PC has experienced significant growth, evolution, and transformation, driven by a deepened understanding of the humanistic and social requirements for aiding patients confronting incurable illnesses and enduring conditions of profound suffering, shifting from studies predominantly confining to patients with advanced cancer.^[52,75]^ In 2015, a systematic review aimed to identify key priorities for future PC research observed that other illnesses had garnered significantly less research attention in comparison to cancer, highlighting the imperative for PC research to extend its focus beyond cancer, encompassing conditions like advanced cardiovascular, neurological, pulmonary, renal, and multiple comorbid diseases.^[51]^

Another aspect that should be highlighted is the rise of publications regarding patients’ refusal of nutrition and artificial nutrition, which alters the predominance of previous publications that were mainly focused on the biomedical aspects and the perceptions, attitudes, knowledge, and experiences of HCPs. Moreover, students should be informed regarding withdrawal and withholding of nutrition/artificial nutrition. This shift indicates a tendency toward a broader approach in the scientific literature, giving higher value to patients and their relatives’ perspectives and emphasizing the importance of understanding patients’ and families’ preferences and wishes, a priority identified by researchers in prior publications.^[51,64]^ The European Society for Clinical Nutrition and Metabolism guideline on ethical aspects of artificial nutrition advocates that ethical principles should guide decisions, particularly in critical and EOF situations, and a competent patient can refuse treatment, even if it may lead to death after receiving adequate information.^[32]^

In the 2017 to 2024 strategic thematic map, family, bereavement, and qualitative research, which were previously absent in the 1994 to 2016 strategic thematic map, appeared with a high density among motor themes. Beyond EOL decisions, which involve scientific, legal, and ethical dimensions, they also encompass profound emotional aspects. What HCPs call nutrition or artificial nutrition, most patients and their relatives perceive as food, with all its cultural, social, physical, and emotional meanings.^[76–81]^ While some PC-EOL patients perceive nutrition as a hope for survival, others cannot feed or choose to stop eating and drinking voluntarily.^[80–83]^ Besides, providing food to those we care about manifests compassion and affection, extending beyond mere nutritional support to establish emotional connections. In this respect, withdrawn artificial nutrition and hydration may be interpreted as an act devoid of love.^[80]^ The significance of food to patients nearing the EOL may be particularly profound, providing a semblance of dignity in the final days and often serving as a primary means of familial bonding and cohesion throughout life before the onset of the illness.^[81]^ Adopting a patient-centric approach to obtaining a dietary history and optimizing menus, incorporating a social component, should constitute a standard practice in the routine nutritional assessment for PC-EOF patients.^[80,84,85]^

### 4.5. Study limitations

Our study has some limitations. First, the search was limited to articles published in WOSCC, and no search was conducted in other databases, such as PubMed and Embase. Second, studies published in recent years might have been overlooked due to limited citations. Third, assessing the quality of articles solely through their citations is challenging when evaluating a large number of papers, and the focus of a bibliometric analysis is not on the individual quality of each article. Finally, bibliometric analyses respond to “how much” and “what” inquiries but do not address “how” or “why” questions. Despite these limitations, our findings may help researchers and HCPs better identify gaps, trends, and new perspectives for future studies on nutrition in PC-EOL.

### 4.6. Gaps and future researcher directions

Despite the gradual evolution of research on nutrition in PC-EOL, significant gaps persist that warrant targeted attention in future investigations. Notably, the predominance of retrospective and cross-sectional studies highlights an urgent need for prospective, longitudinal, and intervention-based research that can generate higher-quality evidence. Moreover, the near absence of publications originating from low- and middle-income countries reveals an inequitable research landscape, which limits the applicability and generalizability of findings to diverse cultural and socioeconomic contexts. Future studies should prioritize the inclusion of underrepresented populations. Additionally, most current research has focused on biomedical aspects of nutrition, often neglecting the psychosocial, cultural, and relational dimensions that profoundly shape patients’ experiences with food at the EOL. Expanding qualitative and mixed-methods research to explore patients’ and families’ perspectives could provide critical insights for the development of more compassionate and person-centered care models.

Furthermore, interdisciplinary collaborations between PC specialists, nutritionists, ethicists, and social scientists remain sparse and should be actively fostered to bridge disciplinary silos. Finally, there is a pressing need for ethical frameworks and innovative study designs that can overcome the methodological challenges inherent in PC-EOL research while ensuring respect for patient’s autonomy and dignity. Addressing these gaps will be essential for advancing both the science and practice of nutritional care in PC-EOL, ultimately improving outcomes and quality of life for this vulnerable population.

## 5. Conclusion

This bibliometric and network analysis reveals that nutrition in PC-EOL remains underrepresented despite its clinical importance. While research output peaked in 2022, the field continues to face ethical, structural, and financial barriers, limiting high-quality prospective studies. Most publications are retrospective or cross-sectional and lack international collaboration. Furthermore, the symbolic and relational aspects of food, central to patient-centered care, are rarely explored. Our findings highlight the need for ethically grounded, humanistic research frameworks and greater inclusion of patient and family perspectives. Strengthening global collaboration, especially in underrepresented regions, is vital for advancing culturally sensitive and interdisciplinary research in PC-EOL nutrition.

## Author contributions

**Conceptualization:** Carlos Álvaro Araújo, Fábio Ferreira Amorim.

**Data curation:** Carlos Álvaro Araújo, Fábio Ferreira Amorim.

**Formal analysis:** Carlos Álvaro Araújo, Fábio Ferreira Amorim.

**Investigation:** Carlos Álvaro Araújo, Rauany Fiuza Braga Pires de Melo, Geisa Sant Anna, Fábio Ferreira Amorim.

**Methodology:** Carlos Álvaro Araújo, Rauany Fiuza Braga Pires de Melo, Fábio Ferreira Amorim.

**Project administration:** Carlos Álvaro Araújo, Fábio Ferreira Amorim.

**Resources:** Carlos Álvaro Araújo, Rauany Fiuza Braga Pires de Melo, Geisa Sant Anna, Fábio Ferreira Amorim.

**Software:** Carlos Álvaro Araújo, Fábio Ferreira Amorim.

**Supervision:** Carlos Álvaro Araújo, Fábio Ferreira Amorim.

**Validation:** Carlos Álvaro Araújo, Rauany Fiuza Braga Pires de Melo, Fábio Ferreira Amorim.

**Visualization:** Carlos Álvaro Araújo, Rauany Fiuza Braga Pires de Melo, Geisa Sant Anna, Fábio Ferreira Amorim.

**Writing** – **original draft:** Carlos Álvaro Araújo, Rauany Fiuza Braga Pires de Melo, Geisa Sant Anna, Fábio Ferreira Amorim.

**Writing** – **review & editing:** Carlos Álvaro Araújo, Rauany Fiuza Braga Pires de Melo, Geisa Sant Anna, Fábio Ferreira Amorim.

## Supplementary Material


