# Supplementary material for: Nutrition in palliative care at the end of life: Bibliometric and network analysis until 2024
Source: Medicine (Baltimore). 2025 Jul 18;104(29):e43381. doi: 10.1097/MD.0000000000043381 (PMC12282806; doi:10.1097/MD.0000000000043381)
Supplement: Supplementary file 1 [file medi-104-e43381-s001.pdf]

Supplementary Table S1. Inclusion and exclusion criteria based on the Population, Context and Concept (PCC) framework

	**Inclusion criteria**	**Exclusion criteria**
Population (P)	Patients in palliative care at the end of life.	Patients in palliative care involving curative therapies
Concept (C)	Nutritional therapy<br>or<br>Patients, caregivers, family members and/or health professionals' perspectives on food/nutrition.	-
Context (C)	Hospital or hospice	-

Supplementary Table S2. Characteristics of publications addressing nutrition in palliative care at the end of life until 2024

Documents, n	314
Authors, n	1504
Co-authors per document, n	5.3
Countries, n	48
International co-authorship, %	13.7
Institutions, n	727
Journals, n	176
Annual growth rate, %	4.7
Average citations per doc, n	23.6
Documents average age, %	9.3

Supplementary Table S3. Publications on nutrition and palliative care until 2024 in terms of number of articles, 2-year moving average and citations

Year	Documents	2-Year moving average	Mean TC per publication	Mean TC per year
1994	4	-	121.5	4.0
1995	2	3.0	37.5	2.0
1996	1	1.5	24.0	1.0
1997	2	1.5	10.0	2.0
1998	3	2.5	55.0	3.0
1999	1	2.0	4.0	1.0
2000	3	2.0	129.0	3.0
2001	3	3.0	32.3	3.0
2002	4	3.5	34.8	4.0
2003	4	4.0	97.2	4.0
2004	5	4,5	39.4	5.0
2005	9	7.0	38.4	9.0
2006	5	7.0	32.8	5.0
2007	4	4.5	29.2	4.0
2008	6	5.0	13.2	6.0
2009	8	4.0	133.6	8.0
2010	5	6.5	48.0	5.0
2011	3	4.0	13.3	3.0
2012	14	8.5	23.1	14.0
2013	6	10.0	24.2	6.0
2014	11	8.5	35.8	11.0
2015	12	11.5	21.7	12.0
2016	17	14.5	31.4	17.0
2017	20	18.5	24.4	20.0
2018	19	19.5	19.0	19.0
2019	12	15.5	8.4	12.00

2020	28	20.0	10.8	28.0
2021	24	26.0	7.8	24.0
2022	35	29.5	5.0	35.0
2023	28	31.5	3.1	28.0
2024	16	22.0	1.0	0.5

TC: total citations

Supplementary Table S4 Publications addressing nutrition in palliative care at the end of life until 2024 by study design and year

Year	Restrospective cohort	Cross-sectional	Narrative Review	Case report/ Case series	Qualitative/ Mixed-Method	Prospective cohort	Systematic/ Scoping/ Integrative/ Umbrella review	Editorial/ Commentary / Letter	Society Guideline/ Society Position Paper	Pre- post-study (quasi-experimental)	Bibliometric/ Visual Network Study	Computational modeling and simulation study	Randomized clinical trial	Total
1994	0	2	0	2	0	0	0	0	0	0	0	0	0	4
1995	0	0	2	0	0	0	0	0	0	0	0	0	0	2
1996	0	0	0	0	0	1	0	0	0	0	0	0	0	1
1997	1	0	1	0	0	0	0	0	0	0	0	0	0	2
1998	0	1	1	0	0	1	0	0	0	0	0	0	0	3
1999	0	0	0	0	0	0	0	0	1	0	0	0	0	1
2000	0	0	1	1	0	0	1	0	0	0	0	0	0	3
2001	0	1	0	0	0	0	0	0	2	0	0	0	0	3
2002	0	1	0	2	0	1	0	0	0	0	0	0	0	4
2003	0	1	1	0	2	0	0	0	0	0	0	0	0	4
2004	2	1	1	0	1	0	0	0	0	0	0	0	0	5
2005	1	2	2	1	1	2	0	0	0	0	0	0	0	9
2006	1	1	0	0	0	2	1	0	0	0	0	0	0	5
2007	1	1	1	1	0	0	0	0	0	0	0	0	0	4
2008	0	4	1	0	0	1	0	0	0	0	0	0	0	6
2009	0	0	4	0	2	2	0	0	0	0	0	0	0	8
2010	2	0	0	2	0	1	0	0	0	0	0	0	0	5

2011	1	0	0	1	1	0	0	0	0	0	0	0	0	3
2012	3	4	2	2	1	1	0	1	0	0	0	0	0	14
2013	1	0	1	1	1	1	1	0	0	0	0	0	0	6
2014	3	1	0	2	1	1	2	1	0	0	0	0	0	11
2015	3	3	1	2	1	1	0	0	1	0	0	0	0	12
2016	3	4	5	0	1	1	1	0	2	0	0	0	0	17
2017	9	4	1	1	1	1	1	2	0	1	0	0	0	20
2018	4	3	5	1	3	2	0	0	0	0	0	0	1	19
2019	3	2	4	0	1	1	1	0	0	0	0	0	0	12
2020	5	9	7	1	1	1	1	1	0	2	0	0	0	28
2021	7	4	4	4	1	1	2	0	0	1	0	0	0	24
2022	5	7	6	1	6	4	3	1	0	1	0	1	0	35
2023	4	4	5	3	4	1	1	2	1	1	1	0	0	28
2024	**5**	**3**	**4**	**2**	**0**	**0**	**1**	**1**	**0**	**0**	**0**	**0**	**0**	16
**Total**	**64**	**63**	**60**	**30**	**29**	**27**	**16**	**9**	**7**	**6**	**1**	**1**	**1**	**314**

Supplementary Table S5. Top 25 countries in terms of number of publications addressing nutrition in palliative care at the end of life and citations until 2024

Ranking	Country	Territory	Publications	Citations
1	US	North America	121	4294
2	UK	Europe	37	872
3	Germany	Europe	21	326
4	Japan	Asia	19	331
5	China	Asia	18	279
6	Italy	Europe	16	368
7	Switzerland	Europe	12	429
8	France	Europe	11	405
9	Canada	North America	11	195
10	Australia	Oceania	11	176
11	Turkey	Europe/Asia	10	36
12	Netherlands	Europe	9	460
13	South Korea	Asia	8	61
14	Spain	Europe	8	47
15	Israel	Asia	7	356
16	Austria	Europe	6	198
17	Brazil	South America	6	11
18	India	Asia	6	5
19	Poland	Europe	5	55
20	Belgium	Europe	4	126
21	Norway	Europe	4	71
22	Denmark	Europe	3	69
23	Singapore	Asia	3	42
24	Finland	Europe	3	35
25	South Africa	Africa	3	21

US: United States; UK: United Kingdom

Supplementary Table S6. Publications addressing nutrition in palliative care at the end of life until 2024 according to the first author's countries

Ranking	Country	Territory	Publications	MCP	SCP	Frequency	MCP ratio
1	US	North America	114	8	106	0.363	0.070
2	UK	Europe	25	4	21	0.080	0.160
3	China	Asia	17	1	16	0.054	0.059
4	Japan	Asia	17	1	16	0.054	0.059
5	Germany	Europe	15	3	12	0.048	0.200
6	Italy	Europe	12	1	11	0.038	0.083
7	Australia	Oceania	10	2	8	0.032	0.200
8	France	Europe	10	0	10	0.032	0.000
9	Turkey	Europe/Asia	10	0	10	0.032	0.000
10	Canada	North America	8	0	8	0.025	0.625
11	Switzerland	Europe	8	5	3	0.025	0.625
12	Spain	Europe	7	0	7	0.022	0.000
13	South Korea	Asia	6	2	4	0.019	0.333
14	Israel	Asia	6	1	5	0.019	0.167
15	Brazil	South America	6	0	6	0.019	0.000
16	Netherlands	Europe	5	2	3	0.016	0.400
17	India	Asia	4	3	1	0.013	0.750
18	Austria	Europe	4	1	3	0.013	0.250
19	Poland	Europe	4	0	4	0.013	0.000
20	Belgium	Europe	3	3	0	0.010	1.000
21	Czech Republic	Europe	2	1	1	0.006	0.500
22	Finland	Europe	2	1	1	0.006	0.500
23	Singapore	Asia	2	1	1	0.006	0.500
24	New Zealand	Oceania	2	0	2	0.006	0.000
25	Norway	Europe	2	0	2	0.006	0.000
26	South Africa	Africa	2	0	2	0.006	0.000
27	Ireland	Europe	1	1	0	0.003	1.000
28	Peru	South America	1	1	0	0.003	1.000
29	Croatia	Europe	1	1	0	0.003	1.000
30	Grenada	Central America	1	1	0	0.003	1.000
31	Chile	South America	1	0	1	0.003	0.000
32	Greece	Europe	1	0	1	0.003	0.000
33	Hungary	Europe	1	0	1	0.003	0.000
34	Indonesia	Asia	1	0	1	0.003	0.000
35	Mexico	Central America	1	0	1	0.003	0.000
36	Portugal	Europe	1	0	1	0.003	0.000
37	Saudi Arabia	Asia	1	0	1	0.003	0.000
38	Uruguay	South America	1	0	1	0.003	0.000

US: United States; UK: United Kingdom

Supplementary Table S7. Publications addressing nutrition in palliative care at the end of life until 2024 according to the corresponding author's countries

Ranking	Country	Territory	Publications	MCP	SCP	Frequency	MCP ratio
1	US	North America	117	11	106	0.373	0.094
2	UK	Europe	26	5	21	0.083	0.192
3	China	Asia	17	1	16	0.054	0.059
4	Japan	Asia	17	1	16	0.054	0.059
5	Germany	Europe	14	2	12	0.045	0.143
6	Italy	Europe	11	0	11	0.035	0.000
7	France	Europe	10	0	10	0.032	0.000
8	Turkey	Europe/Asia	10	0	10	0.032	0.000
9	Australia	Oceania	9	1	8	0.029	0.111
10	Switzerland	Europe	8	5	3	0.025	0.625
11	Canada	North America	8	0	8	0.025	0.000
12	Spain	Europe	7	0	7	0.022	0.000
13	South Korea	Asia	6	2	4	0.019	0.333
14	Israel	Asia	6	1	5	0.019	0.167
15	Brazil	South America	6	0	6	0.019	0.000
16	Netherlands	Europe	5	2	3	0.016	0.400
17	India	Asia	4	3	1	0.013	0.750
18	Austria	Europe	4	1	3	0.013	0.250
19	Poland	Europe	4	0	4	0.010	0.000
20	Belgium	Europe	3	3	0	0.006	1.000
21	Czech Republic	Europe	2	1	1	0.006	0.500
22	Finland	Europe	2	1	1	0.006	0.500
23	Singapore	Asia	2	1	1	0.006	0.500
24	New Zealand	Oceania	2	0	2	0.006	0.000
25	Norway	Europe	2	0	2	0.006	0.000
26	South Africa	Africa	2	0	2	0.006	0.000
27	Ireland	Europe	1	1	0	0.003	1.000
28	Peru	South America	1	1	0	0.003	1.000
29	Chile	South America	1	0	1	0.003	0.000
30	Greece	Europe	1	0	1	0.003	0.000
31	Hungary	Europe	1	0	1	0.003	0.000
32	Indonesia	Asia	1	0	1	0.003	0.000
33	Mexico	Central America	1	0	1	0.003	0.000
34	Portugal	Europe	1	0	1	0.003	0.000
35	Saudi Arabia	Asia	1	0	1	0.003	0.000
36	Uruguay	South America	1	0	1	0.003	0.000

US: United States; UK: United Kingdom

Supplementary Table S8. Top 10 authors in terms of publications addressing nutrition in palliative care at the end of life, citations and local impact until 2024

Ranking	Author	Organization	Country	Publications	Total Citations	H-index	G-index	M-index
1	Mitchell SL	Havard University	US	5	1174	5	5	0.263
2	Bruera E	University of Texas	US	5	125	5	5	0.357
3	Quill TE	University of Rochester	US	5	148	5	5	0.192
4	Yager J	University of Colorado	US	5	148	4	5	0.250
5	Morita T	Seirei Mikatahara General Hospital	Japan	5	116	4	5	0.400
6	Deliens L	Vrije Universiteit Brussel	Belgium	4	126	4	4	0.190
7	Tsuneto S	Kyoto University	Japan	4	88	3	4	0.300
8	Lorenz KA	Stanford University	US	4	87	4	4	0.182
9	Gaudiani JL	University of Colorado	US	4	66	3	4	0.214
10	Fringer A	Witten/Herdecke University	Germany	4	36	4	4	0.500

US: United States

Supplementary Table S9. Main characteristics of top 10 journals in terms of the number of publications addressing nutrition in palliative care at the end of life and citations until 2024

Ranking	Journal	Publications	Total Citations	H-index	G-index	M-index	Year of First Publication
1	Journal of Pain and Symptom Management	20	359	10	18	0.417	2002
2	Journal of Palliative Medicine	18	203	9	14	0.450	2006
3	American Journal of Hospice & Palliative Medicine	12	135	6	11	0.462	2013
4	Palliative Medicine	10	306	8	10	0.258	1995
5	BMJ Supportive & Palliative Care	10	73	3	8	0.300	2016
6	Supportive Care in Cancer	9	231	7	9	0.226	1995
7	BMC Palliative Care	8	50	4	6	0.400	2016
8	Journal of Palliative Care	8	94	5	8	0.172	1997
9	Journal of Eating Disorders	6	100	5	6	1.250	2022
10	Journal of the American Geriatrics Society	6	282	6	6	0.188	1994

Supplementary Table S10. Main characteristics of top 14 institutions in terms of the number of publications addressing nutrition in palliative care at the end of life and citations until 2024

Ranking	Journal	Country	Territory	Publications	Total Citations
1	Harvard University	US	North America	8	1576
2	University of Colorado	US	North America	8	208
3	University of Texas Houston	US	North America	8	190
4	Oregon Health & Science University	US	North America	6	244
5	Johns Hopkins University	US	North America	6	146
6	Pennsylvania State University	US	North America	5	1124
7	Univerversity of Rochester	US	North America	5	478
8	University of Pennsylvania	US	North America	5	197
9	Icahn School of Medicine at Mount Sinai	US	North America	5	130
10	Seirei Mikatahara General Hospital	Japan	Asia	5	116
11	University of Toronto	Canada	North America	5	92
12	University of Washington	US	North America	5	92
13	ZHAW Zurich University of Applied Sciences	Switzerland	Europe	5	82
14	Duke University	US	North America	5	74

US: United States

Supplementary Table S11. Top 20 publications addressing nutrition in palliative care at the end of life ranking in term of citations until 2024

Ranking	Authors (year)	Journal	Global Citations	Local Citations	WOS Category
1	Mitchell et al. (2009)[27]	New England Journal of Medicine	877	8	Medicine, General & Internal
2	McCann et al. (1994)[28]	Journal of the American Medical Association	336	15	Medicine, General & Internal
3	Eddy et al. (2017)[46]	Journal of Clinical Psychiatry	281	4	Psychology, Clinical; Psychiatry
4	Nitenberg et al. (2000)[29]	Critical Reviews in Oncology/Hematology	271	1	Oncology; Hematology
5	Druml et al. (2016)[31]	Clinical Nutrition	178	14	Nutrition & Dietetics
6	Ganzini et al. (2003)[30]	New England Journal of Medicine	150	20	Medicine, General & Internal
7	Olson et al. (2014)[33]	JAMA Surgery	130	3	Surgery
8	Caccialanza et al. (2016)[34]	Journal of Cancer	107	1	Oncology
9	Uster et al. (2018)[35]	Clinical Nutrition	105	2	Nutrition & Dietetics
10	Mills et al. (1994)[36]	British Medical Journal	99	0	Medicine, General & Internal
11	Tjia et al. (2014)[37]	JAMA Internal Medicine	93	0	Medicine, General & Internal
12	Angus et al. (2003)[38]	American Journal of Gastroenterology	92	0	Gastroenterology & Hepatology
13	Brown et al. (2013)[39]	Palliative Medicine	91	2	Health Care Sciences & Services; Public, Environmental & Occupational Health; Medicine, General & Internal
14	Quill et al. (2000)[40]	Annals of Internal Medicine	89	6	Medicine, General & Internal
15	Mitchell et al. (2010)[41]	Journal of Pain and Symptom Management	89	5	Health Care Sciences & Services; Medicine, General & Internal; Clinical Neurology
16	Reid et al. (2009)[42]	International Journal of Nursing Studies	89	1	Nursing
17	Carlson et al. (2012)[43]	Health Affairs	88	0	Health Care Sciences & Services; Health Policy & Services
18	Kayser-Jones et al. (2003)[44]	Gerontologist	88	0	Gerontology
19	Pothuri et al. (2005)[45]	Gynecologic Oncology	83	3	Oncology; Obstetrics & Gynecology
20	Kiely et al. (2010)[46]	Journal of the American Geriatrics Society	82	0	Geriatrics & Gerontology; Gerontology

WOS: Web of Science

Supplementary Table S12. Countries' collaboration regarding publications addressing nutrition in palliative care at the end of life until 2024

Node	Cluster	BC	CC	PageRank
UK	4	254.479	0.223	0.115
US	4	233.504	0.021	0.097
India	3	42.908	0.016	0.038
Italy	1	18.031	0.017	0.040
Switzerland	2	14.701	0.018	0.065
Netherlands	1	12.117	0.016	0.053
Denmark	1	11.021	0.017	0.065
Austria	2	7.828	0.016	0.030
South Korea	5	6.594	0.015	0.029
Norway	1	4.957	0.016	0.027
Germany	2	4.670	0.017	0.056
Japan	5	3.297	0.015	0.029
Finland	1	2.403	0.015	0.024
Canada	2	2.059	0.016	0.025
Belgium	1	1.711	0.015	0.037
Sweden	1	0.484	0.012	0.023
Israel	2	0.236	0.014	0.019
Czech Republic	6	0.000	1.000	0.027
Slovakia	6	0.000	1.000	0.027
Australia	4	0.000	0.015	0.013
Ireland	4	0.000	0.015	0.010
China	5	0.000	0.014	0.022
Nepal	3	0.000	0.014	0.017
Singapore	5	0.000	0.014	0.016
Poland	1	0.000	0.014	0.016
France	1	0.000	0.014	0.010
Pakistan	3	0.000	0.014	0.010
Croatia	3	0.00	0.013	0.011
Peru	2	0.000	0.013	0.010
Estonia	12	0.000	0.013	0.007
Thailand	13	0.000	0.013	0.007
Spain	7	0.000	0.014	0.014
Argentina	7	0.000	0.013	0.012
South Africa	9	0.000	0.012	0.007
Malawi	10	0.000	0.012	0.007
Botswana	11	0.000	0.012	0.007
Saudi Arab	8	0.000	0.010	0.008

BC: betweenness centrality; CC: closeness centrality; UK: United Kingdom; US: United States

Supplementary Table S13. Institutions' collaboration in publications addressing nutrition in palliative care at the end of life until 2024

Node	Cluster	BC	CC	PageRank
University of North Carolina	1	121.000	0.018	0.025
Duke University	3	114.000	0.016	0.031
Johns Hopkins University	1	104.500	0.016	0.035
Icahn School of Medicine at Mount Sinai	1	91.500	0.017	0.038
Harvard University	10	81.000	0.016	0.043
University of Pennsylvania	1	52.500	0.014	0.021
Yale University	1	46.000	0.013	0.019
University of Chicago	1	25.500	0.013	0.014
University of Zurich	3	24.000	0.012	0.029
University of Copenhagen	3	24.000	0.012	0.029
Medical College of Wisconsin	4	24.000	0.012	0.017
University of Colorado	7	24.000	0.012	0.024
University of Rochester	6	24.000	0.010	0.019
University of Texas Houston	9	24.000	0.010	0.022
National Taiwan University	2	6.000	0.111	0.017
Seirei Mikatahara General Hospital	2	2.411	0.125	0.035
University of Turin	5	2.000	0.333	0.035
Kyoto University	2	1.982	0.125	0.033
Tohoku University	2	1.607	0.125	0.029
University of Washington	8	0.000	1.000	0.022
Veteran Affairs Puget Sound Health Care System Seattle	8	0.000	1.000	0.022
Emory University	11	0.000	1.000	0.022
Lancaster University	11	0.000	1.000	0.022
Taipei Veterans General Hospital	2	0.000	0.067	0.006
Necker-Enfants Malades Hospital	12	0.000	0.500	0.024
Hôpital Raymond-Poincaré	12	0.000	0.500	0.024
Institut Gustave Roussy	12	0.000	0.500	0.019
Molinette Hospital	5	0.000	0.250	0.026
University of Milan	5	0.000	0.250	0.018
Radboud University Nijmegen	5	0.000	0.200	0.011
University College London	2	0.000	0.100	0.012
Kobe University	2	0.000	0.091	0.023
Osaka City General Hospital	2	0.000	0.091	0.021
Pennsylvania State University	10	0.000	0.012	0.025
Brown University	10	0.000	0.012	0.025
University of Toronto	1	0.000	0.012	0.022
Sunnybrook Health Sciences Centre	1	0.000	0.012	0.022
Cardiff University	3	0.000	0.012	0.022
University of Edinburgh	3	0.000	0.012	0.022
ZHAW Zurich University of Applied Sciences	3	0.000	0.010	0.008

Technical University of Munich	3	0.000	0.010	0.008
Gaudiani Clinic	7	0.000	0.009	0.019
University of Wisconsin	4	0.000	0.009	0.011
Oregon Health & Science University	6	0.000	0.008	0.011
University of Houston	9	0.000	0.008	0.016

BC: betweenness centrality; CC: closeness centrality

Supplementary Table S14. High-frequency authors' keywords on publications addressing nutrition in palliative care at the end of life until 2024

Keyword	Frequency
Palliative care	155
Cancer	99
End of life	79
Hospice	68
Nutrition	68
End of life care	51
Advanced cancer	31
Hospice care	28
Ethics	25
Parenteral nutrition	23
Hydration	22
Refusal	22
Quality of life	21
Terminal illness	21
Dying	18
Eating disorders	17
Enteral nutrition	17
Gastrointestinal obstruction	17
Obstruction	17
Advance care planning	16
Caregivers	16
Dementia	16
Home care	16
Life-sustaining	16
Elderly	15
Anorexia nervosa	14
Bowel obstruction	14
Gastrointestinal	14
Hospital	14
Life-sustaining treatments	14
Symptoms	14
Voluntarily stopping eating and drinking	14
Advanced dementia	13
Nurses	13
Sedation	13
Family	12
Nutritional support	12
Cachexia	11

Intensive care	11
Intensive care units	11

Supplementary Table S15. Viewpoints of the top 20 publications addressing nutrition in palliative care at the end of life ranking in terms of citations until 2024

Ranking	Authors (year)	Title	Article type	Viewpoint	Authors' keyword cluster	Nutrition in palliative care as a core element?
1	Mitchell et al. (2009)[27]	The clinical course of advanced dementia	Prospective cohort study	Eating problems are a frequent complication in people with advanced dementia (85.8%), highlighting nutrition's critical role in their care.	End-of-life Care in Advanced Dementia	No
2	McCann et al. (1994)[28]	Comfort care for terminally ill patients. The appropriate use of nutrition and hydration	Case series study	Most terminally ill patients, especially those with cancer, did not experience significant hunger. Even for those who did, only small quantities of food were needed for relief. Similarly, minimal fluid intake was effective in alleviating thirst and dry mouth. These findings suggest that fulfilling specific and minimal patient requests for nutrition and hydration might be adequate, emphasizing a conservative approach to nutrition in end of life care.	Comprehensive End-of-Life Care	Yes
3	Eddy et al. (2017)[29]	Recovery from anorexia nervosa and bulimia nervosa at 22-year follow-up	Prospective cohort study	Palliative care management has been suggested when the duration of anorexia nervosa or bulimia nervosa exceeds a decade. However, the recovery from anorexia nervosa continued long term, arguing against the implementation of palliative care for most patients with eating disorders.	Palliative Care in Anorexia Nervosa and Eating Disorders	No
4	Nitenberg et al. (2000)[30]	Nutritional support of the cancer patient: issues and	Narrative review	Nutritional support in palliative care should be based on the potential risks and	Advanced Cancer	No

		dilemmas		benefits of enteral and parenteral nutrition and the patients' and families' wishes.	Nutritional Support	
5	Druml et al. (2016)[32]	ESPEN guideline on ethical aspects of artificial nutrition and hydration	Guideline/Society position article	Artificial nutrition and hydration are medical interventions that necessitate ethical considerations and patient consent, considering the patient's condition and cultural differences. Ethical principles should guide decisions, particularly in critical and end of life situations. If the risks and burdens of a therapy outweigh the benefits, the physician should withhold the therapy. A competent patient can refuse treatment, even if it may lead to death after receiving adequate information.	Ethical Decision-Making in End-of-Life Choices	Yes
6	Ganzini et al. (2003)[31]	Nurses' experiences with hospice patients who refuse food and fluids to hasten death	Cross-sectional (online survey)	According to nurses' perspectives, the decision to refuse food and fluids is associated with the patient's readiness to die and their perspectives on the means of life. Usually, they are elderly and have a peaceful death within two weeks after stopping food and fluids.	Ethical Decision-Making in End-of-Life Choices	Yes
7	Olson et al. (2014)[33]	Palliative surgery for malignant bowel obstruction from carcinomatosis: a systematic review	Systematic review	Palliative surgery can benefit patients in the resumption of a diet. However, it has a high complication rate and mortality, leading to a substantial increase in the hospital length of stay concerning the patient's remaining survival time. It is essential to consider the challenges and benefits of surgery regarding nutrition and dietary habits in terminal illnesses and present realistic goals and limitations of surgery, particularly concerning	Gastrointestinal obstruction	Yes

				nutritional aspects.		
8	Caccialanza et al. (2016)[34]	Nutritional support in cancer patients: a position paper from the Italian Society of Medical Oncology (AIOM) and the Italian Society of Artificial Nutrition and Metabolism (SINPE)	Guideline/Society position article	Malnutrition has a critical role in cancer patients. Nutritional support may be integrated into palliative care programs, acknowledging the importance of addressing nutritional needs in end of life care.	Advanced Cancer Nutritional Support	No
9	Uster et al. (2018)[35]	Effects of nutrition and physical exercise intervention in palliative cancer patients: A randomized controlled trial	Randomized controlled trial	Combining nutrition and physical exercise improves the patients' overall well-being and nutritional status, shedding light on the potential benefits of a multimodal approach in palliative care. The emphasis on adequate protein intake and symptom reduction, like nausea and vomiting, showcases the importance of tailoring interventions to palliative cancer patients' specific needs and challenges.	Advanced Cancer Nutritional Support	Yes

10	Mills (1994)[36]	Care of dying patients in hospital	Case Series	In the context of care for dying patients in hospitals, there were deficiencies in providing elemental care related to nutrition, such as lack of assistance with eating and inadequate attention to thirst. Improving end of life care is fundamental, including better attention to nutritional aspects, to enhance the quality of healthcare for dying patients.	Comprehensive End-of-Life Care	No
11	Tjia et al. (2014)[37]	Use of medications of questionable benefit in advanced dementia	Cross-sectional	Among the 5406 nursing home residents, 39.1% had nutritional problems (≥ 25% of meals uneaten, complaints of hunger or complaints of taste), 15.4% had a feeding tube, 74.3% had oral problems (chewing problems, swallowing problems, or mouth pain), and 53.9% used at least one questionably beneficial medication. Residing in a facility with a high prevalence of feeding tubes (> 10% of facility residents) was associated with a greater likelihood of being given questionably beneficial medications compared with residents in nursing homes where the prevalence of feeding tubes was 0% to 5%. Nursing home residents with oral problems and feeding tubes were less likely to be prescribed at least one questionably beneficial medication.	End-of-life Care in Advanced Dementia	No
12	Angus et al. (2003)[38]	The percutaneous endoscopic gastrostomy tube: medical and ethical issues in placement	Narrative review	Percutaneous endoscopic gastrostomy tube placement should be assessed critically to ensure it serves the patient's best interests, providing nutrition and significantly contributing to their overall well-being, symptom relief, and quality of	Gastrointestinal obstruction	No

				life. Patients and caregivers must fully understand the implications, benefits, and potential challenges of percutaneous endoscopic gastrostomy tube placement. Informed decision-making empowers patients to actively participate in their care, aligning with the palliative care approach, which often emphasizes patient autonomy and involvement in decision-making.		
13	Brown et al. (2013)[39]	Prognostic indicators of 6-month mortality in elderly people with advanced dementia: A systematic review	Systematic review	Few studies have comprehensively addressed potential prognosticators for advanced dementia. The most common predictive variables identified in the examined literature were related to nutrition/nourishment or eating habits, followed by increased risk on dementia severity scales and comorbidities.	End-of-life Care in Advanced Dementia	No
14	Quill et al. (2000)[40]	Palliative treatments of last resort: choosing the least harmful alternative	Case series	Nutritional decisions should align with the patient's values and goals of care, especially in the challenging context of end of life care, coordinating with a patient-centered approach in palliative care. If provided, nutritional support should be in harmony with the patient's wishes and contribute to their overall comfort and well-being.	Comprehensive End-of-Life Care	No
15	Mitchell et al. (2010)[41]	Prediction of 6-month survival of nursing home residents with advanced dementia using ADEPT vs hospice eligibility guidelines	Retrospective cohort study	Among the 215 nursing home residents with advanced dementia, 35.5% were at a FAST stage 7c (severe dementia and can no longer walk), 41.6% had insufficient oral intake, 8.3% had a body mass index of less than 18.5, and 11.7% had recent	End-of-life Care in Advanced Dementia	No

				weight loss. As a continuous variable, the Advanced Dementia Prognostic Tool (ADEPT), which includes insufficient oral intake or tube feeding with impaired nutritional status as one of its components, had an area under the receiver operating characteristic curve (AUROC) for 6-month mortality prediction of 0.67 (95% IC: 0.62–0.72). A cut point of more than 11.0 achieved the highest AUROC (0.63; 95% CI: 0.58–0.68) with a sensitivity of 55.0% (95% CI, 45.2–64.4) and specificity of 71.3% (95% CI, 67.1–75.3).		
16	Reid et al. (2009)[42]	The experience of cancer cachexia: A qualitative study of advanced cancer patients and their family members	Qualitative study (unstructured interview with interpretative phenomenological analysis)	Cachexia in advanced cancer profoundly impacts patients and families across physical, psychological, and social domains. Patients view eating as vital for maintaining life. Participants did not understand appetite alterations in cachexia, causing difficulties for patients and families. Patients acutely feel the severe implications of cachexia on their appearance and life. Family members often equate increased food intake with hope for prolonged life, revealing a lack of awareness of cachexia's severity. Accurate information is crucial to mitigate conflicts, particularly concerning dietary intake, emphasizing the need for education.	Advanced Cancer Nutritional Support	Yes
17	Carlson et al. (2012)[43]	Hospices' enrollment policies may contribute to underuse of hospice care in the United States	Cross-sectional (Online survey)	There are critical aspects of hospice care related to access barriers for patients with specific medical needs, including nutrition-related needs like total parenteral	Comprehensive End-of-Life Care	No

				nutrition. This finding indicates a potential gap in palliative care accessibility, suggesting that policy changes might be necessary to enhance access for patients with diverse medical requirements, including nutritional support.		
18	Kayser-Jones et al. (2003)[44]	Factors that influence end of life care in nursing homes: The physical environment, inadequate staffing, and lack of supervision	Qualitative study (in-depth interviews and event analysis)	Nursing home residents often did not receive elemental care, such as bathing, oral hygiene, adequate food and fluids, and repositioning in nursing homes. They often stated that the nursing home residents were thirsty and frequently asked for something to drink. However, although receiving inadequate fluids was a significant problem for nursing home residents, their families' greatest fear was that they would not be fed since residents often had a good appetite. Families felt obligated to stay in the nursing home to feed them in the weeks and months before death.	Comprehensive End-of-Life Care	No
19	Pothuri et al. (2005)[45]	Percutaneous endoscopic gastrostomy tube placement in patients with malignant bowel obstruction due to ovarian carcinoma	Retrospective cohort study	Percutaneous endoscopic gastrostomy tube placement is technically feasible and safe in the palliative care of patients with advanced ovarian carcinoma, allowing most patients to have end of life care at home or in an inpatient hospice. However, it is essential to have individualized approaches based on factors such as age and liver metastases. After percutaneous endoscopic gastrostomy tube placement,	Comprehensive End-of-Life Care	Yes

				total parenteral nutrition did not significantly change the patient's prognosis.		
20	Kiely et al. (2010)[46]	Hospice use and outcomes in nursing home residents with advanced dementia	Prospective cohort study	Eating problems (weight loss, swallowing problems, chewing problems, spitting food, and refusal to eat) were independently associated with hospice referral. Improving care and comfort during the last days of life through hospice utilization involves a holistic approach encompassing nutrition, symptom management, and psychological support.	Advanced Cancer Nutritional Support	No
