# Supplementary figures and images for: Nutrition in palliative care at the end of life: Bibliometric and network analysis until 2024
Source: Medicine (Baltimore). 2025 Jul 18;104(29):e43381. doi: 10.1097/MD.0000000000043381 (PMC12282806; doi:10.1097/MD.0000000000043381)

## Slide 1
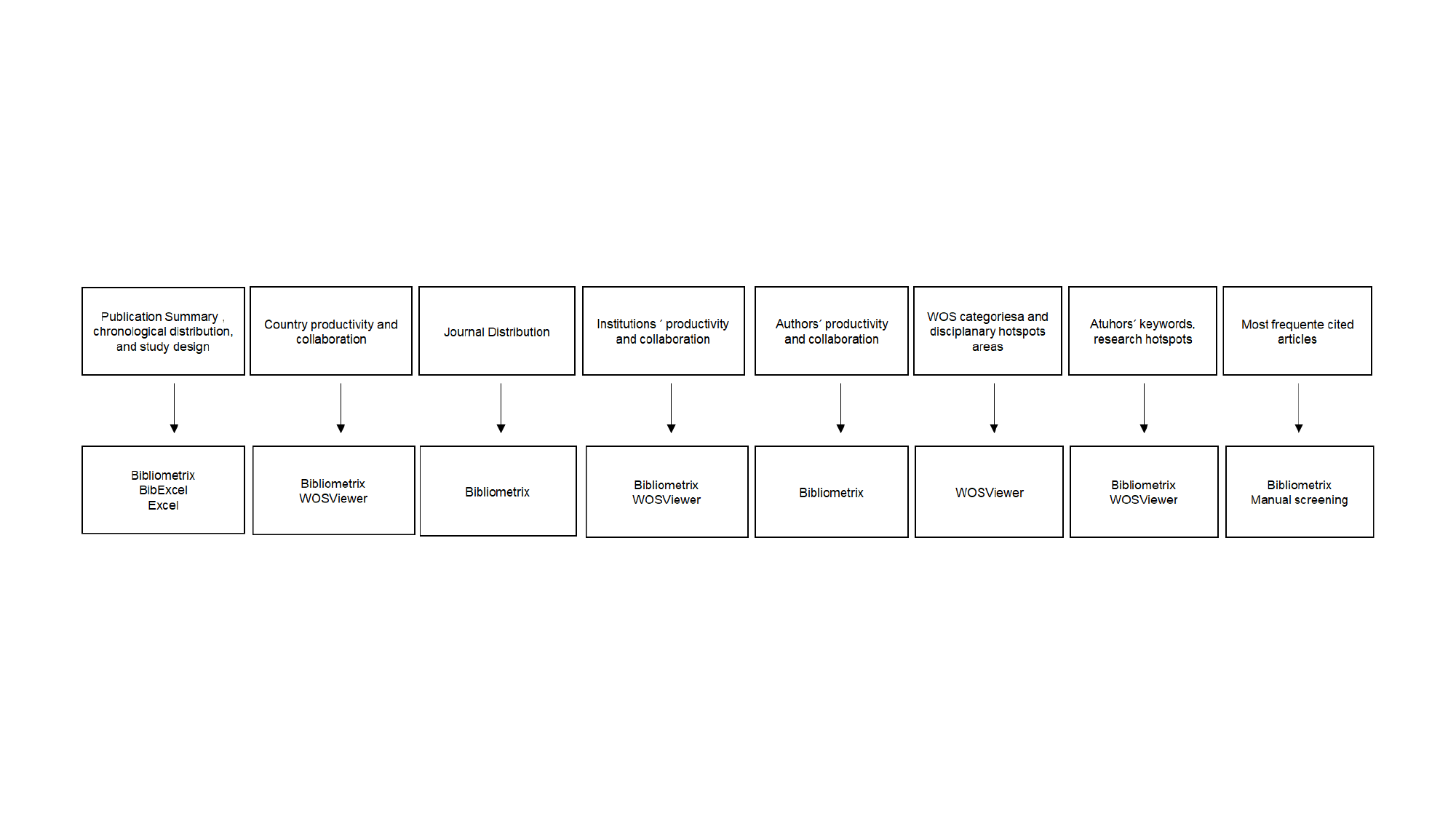

## Slide 2
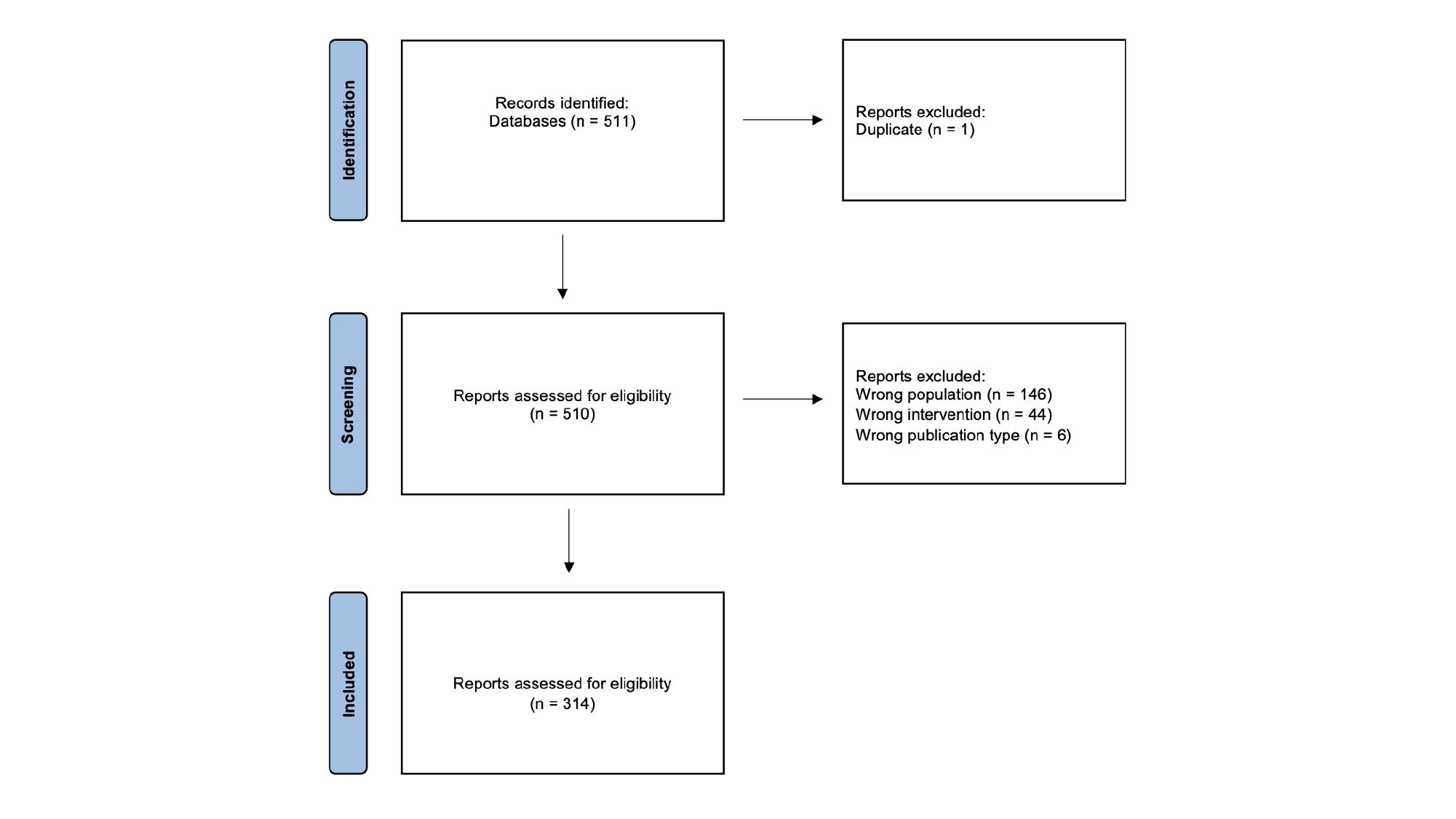

## Slide 3
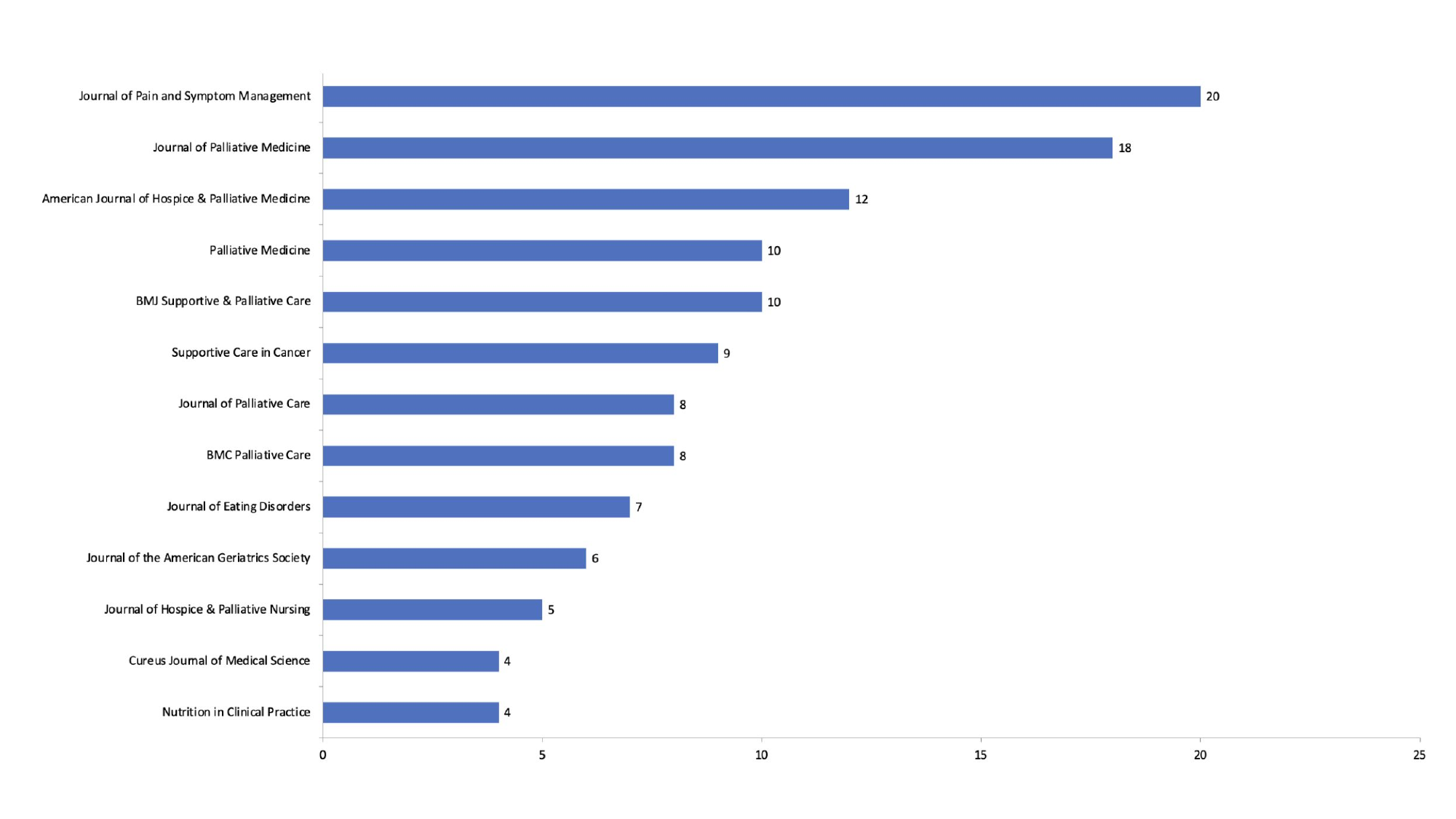

## Slide 4
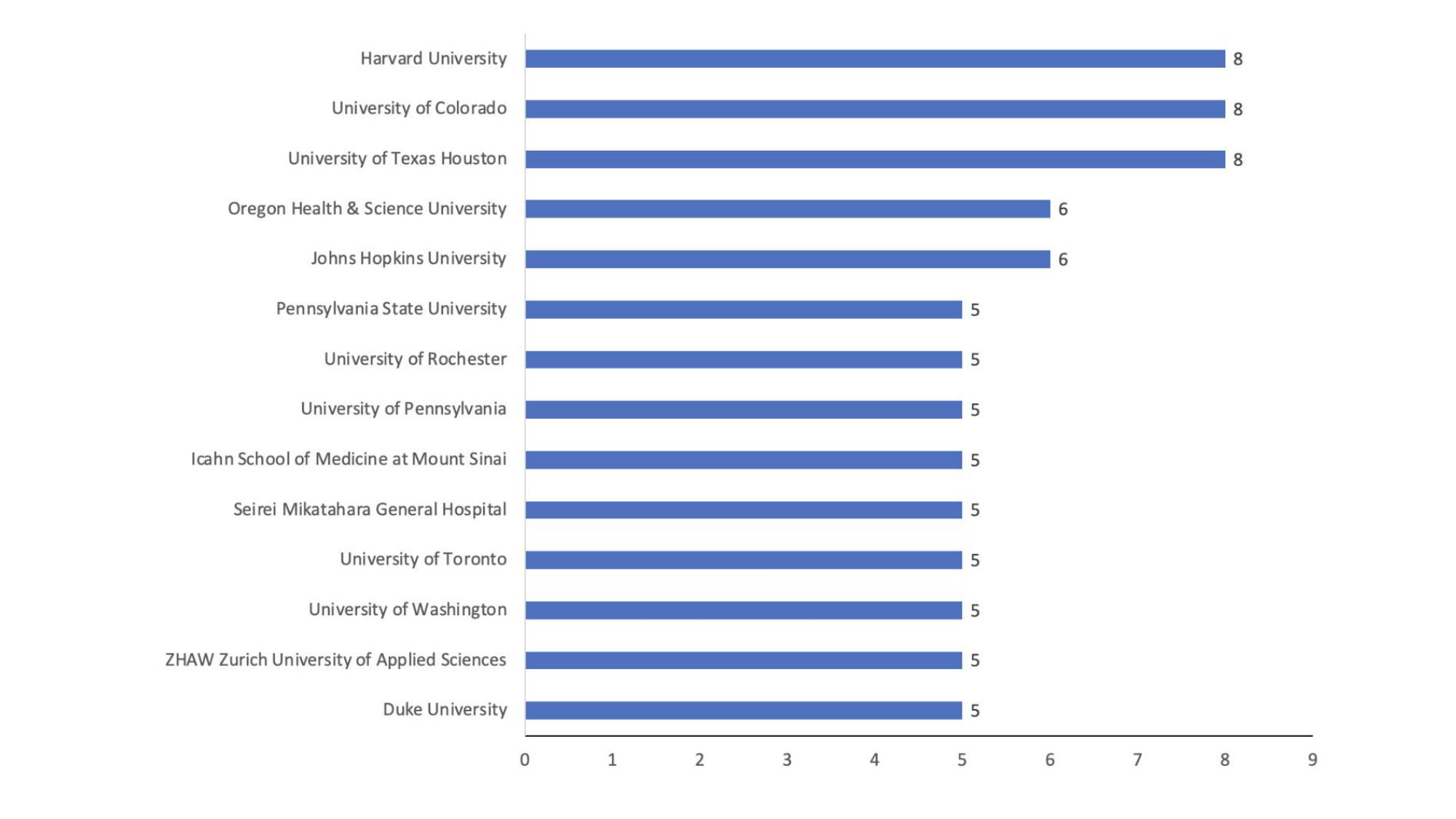

## Slide 5
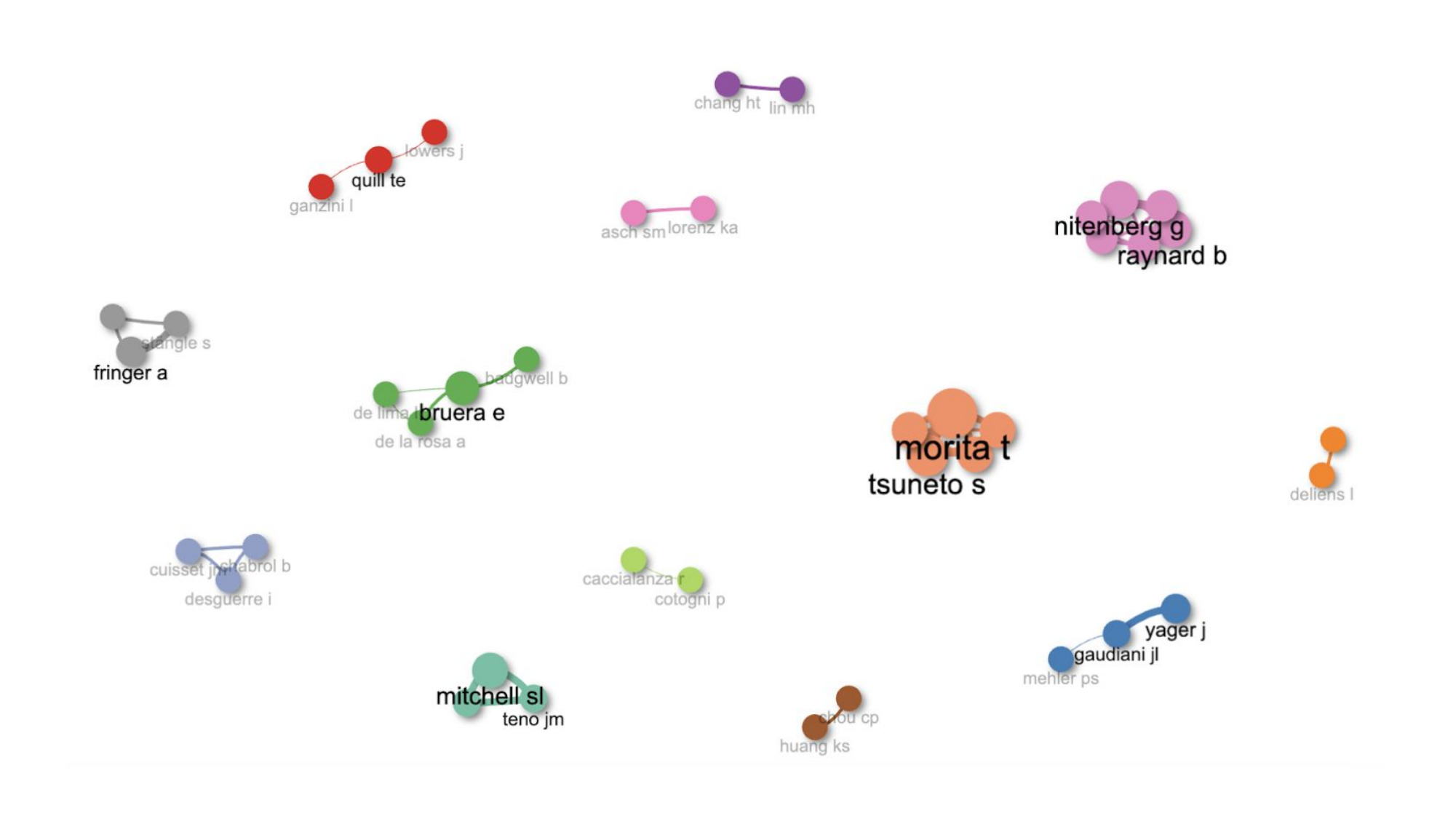

## Slide 6
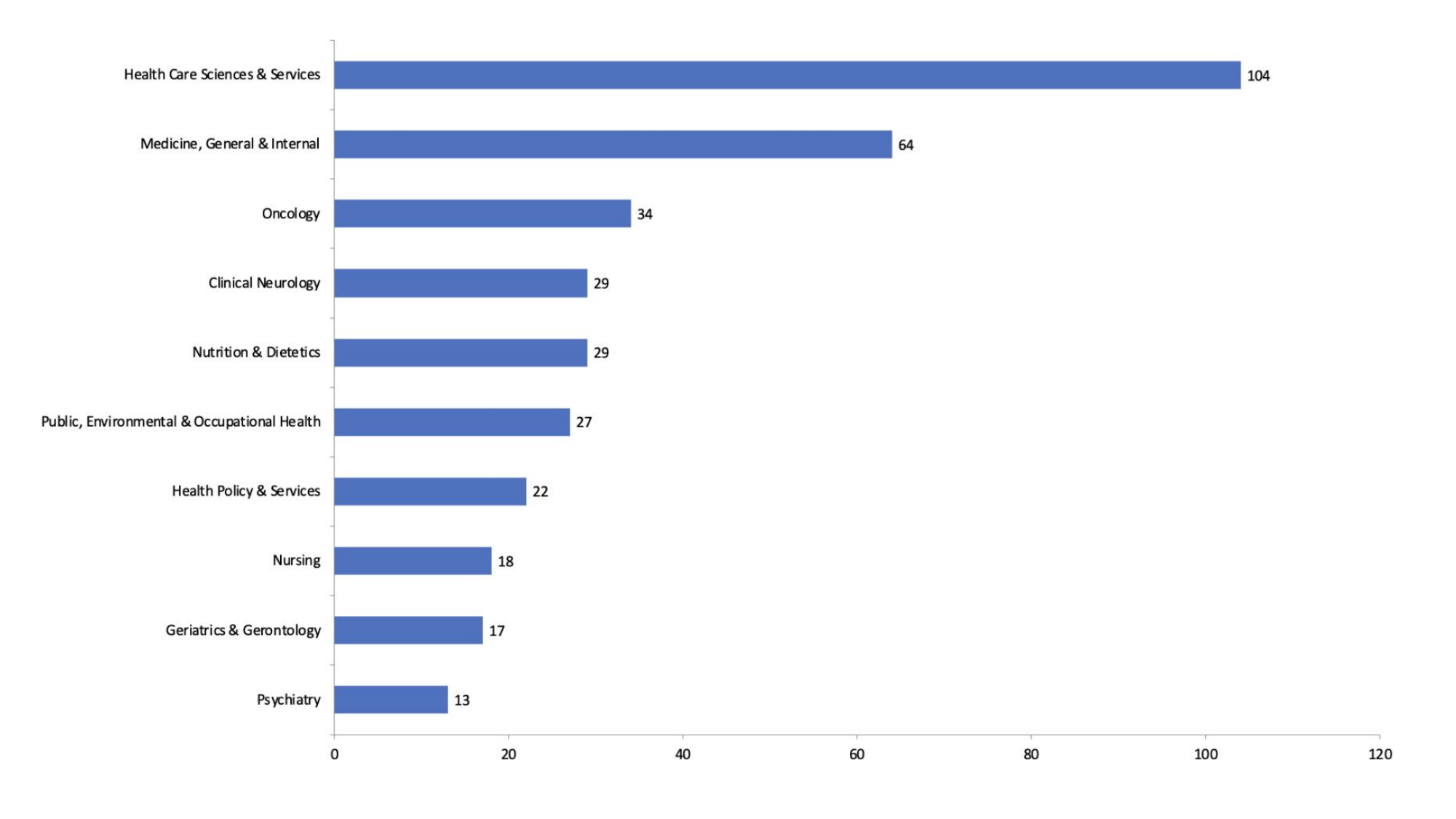

## Slide 7
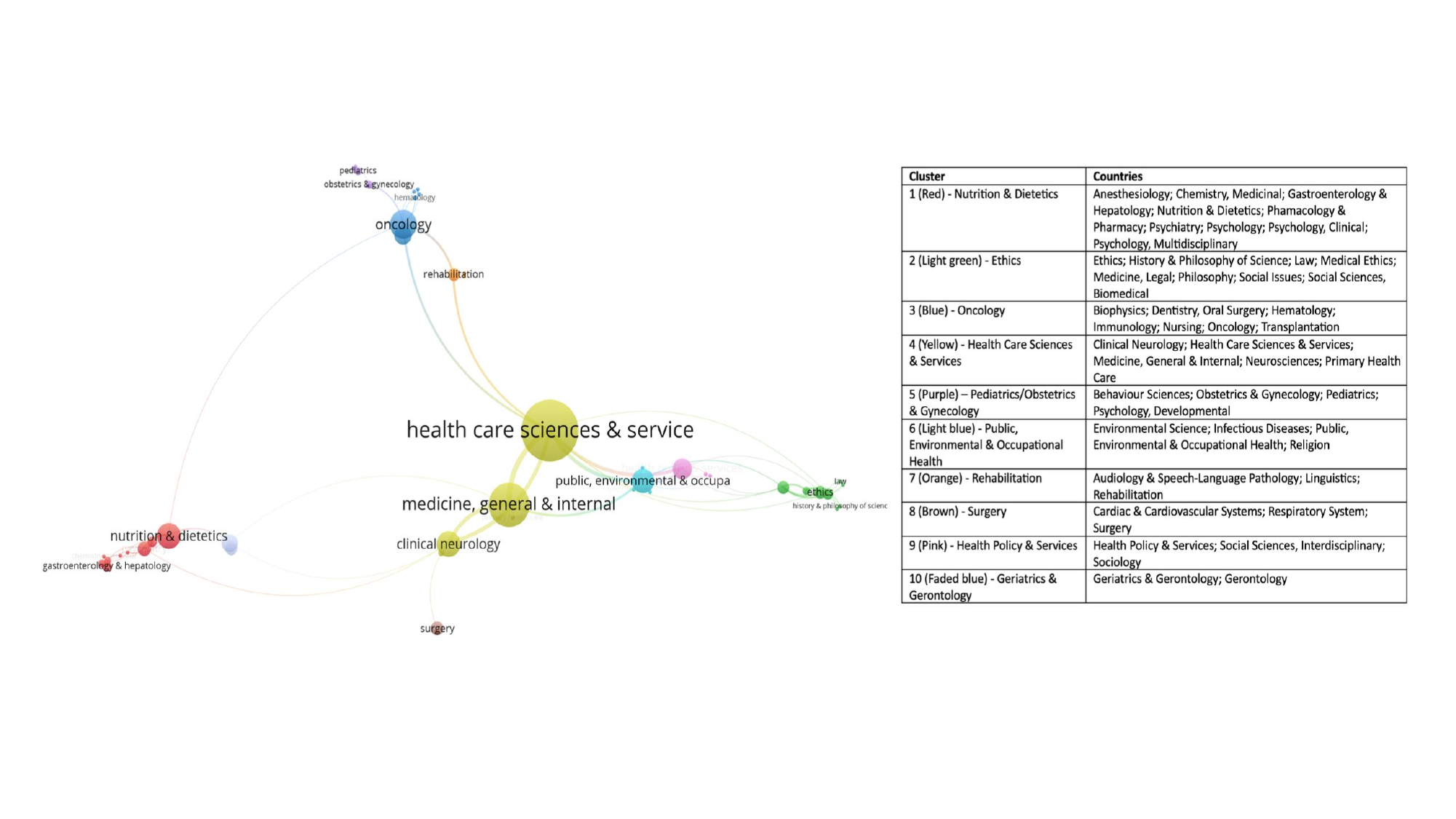

## Slide 8
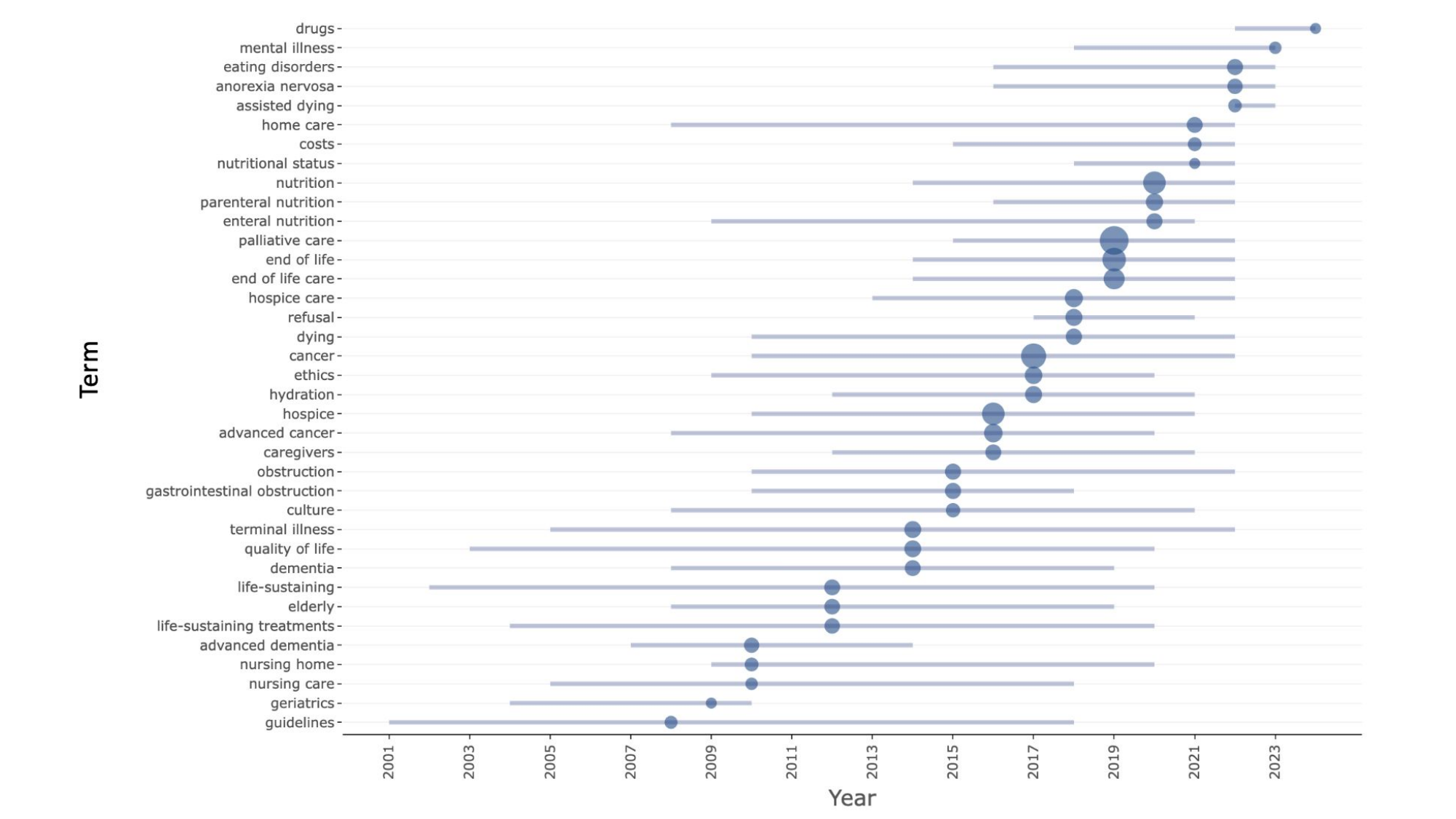

Supplement: Supplementary file 2 [file medi-104-e43381-s002.pptx]
